# Reverse Ordered Sequential Mechanism for Lactoperoxidase with Inhibition by Hydrogen Peroxide

**DOI:** 10.3390/antiox10111646

**Published:** 2021-10-20

**Authors:** Kellye Cupp-Sutton, Michael T. Ashby

**Affiliations:** Department of Chemistry and Biochemistry, University of Oklahoma, Norman, OK 73019, USA; kellye.a.cupp-1@ou.edu

**Keywords:** lactoperoxidase, thiocyanate, kinetics, mechanism

## Abstract

Lactoperoxidase (LPO, Fe^III^ in its resting state in the absence of substrates)—an enzyme secreted from human mammary, salivary, and other mucosal glands—catalyzes the oxidation of thiocyanate (SCN^−^) by hydrogen peroxide (H_2_O_2_) to produce hypothiocyanite (OSCN^−^), which functions as an antimicrobial agent. The accepted catalytic mechanism, called the halogen cycle, comprises a two-electron oxidation of LPO by H_2_O_2_ to produce oxoiron(IV) radicals, followed by O-atom transfer to SCN^−^. However, the mechanism does not explain biphasic kinetics and inhibition by H_2_O_2_ at low concentration of reducing substrate, conditions that may be biologically relevant. We propose an ordered sequential mechanism in which the order of substrate binding is reversed, first SCN^−^ and then H_2_O_2_. The sequence of substrate binding that is described by the halogen cycle mechanism is actually inhibitory.

## 1. Introduction

**Figure 1:**
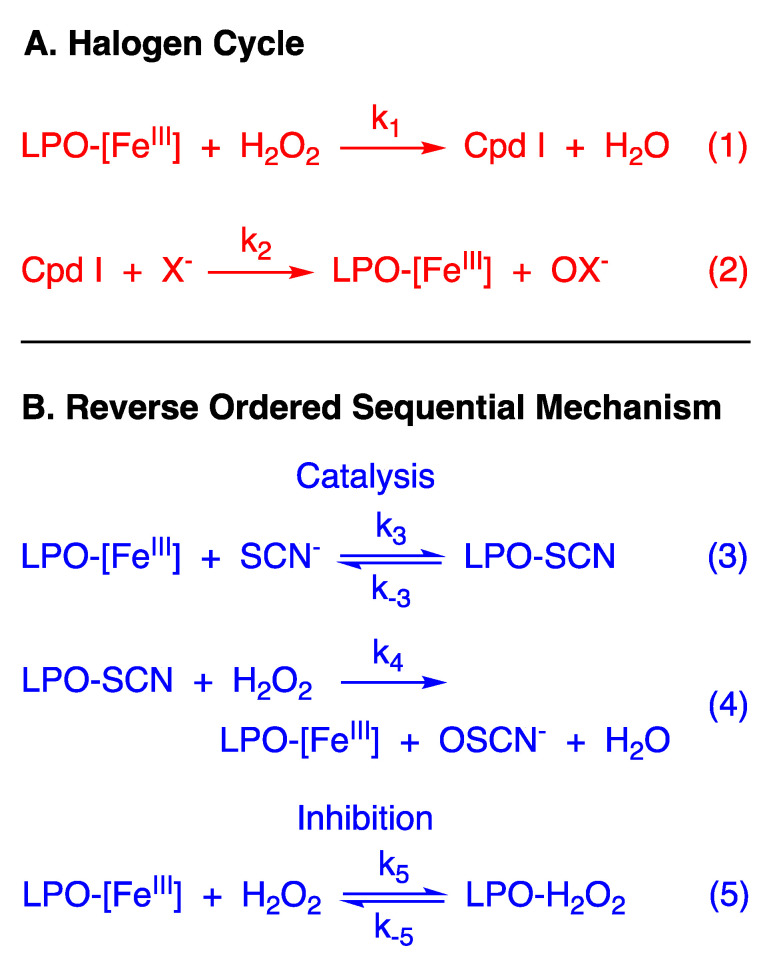
(**A**) The literature “Halogen Cycle” mechanism of the LPO-catalyzed oxidation of halides and pseudohalide. (**B**) Proposed (reverse) ordered sequential kinetic mechanism of the LPO-catalyzed oxidation of thiocyanate and inhibition by tight binding of hydrogen peroxide.

Secretory proteins play essential roles in human host defense. Perhaps the most widely studied secretory protein is lactoperoxidase (LPO, EC 1.11.1.7) [1]. LPO is a heme oxidoreductase (Fe^III^ in its resting state) that catalyzes the oxidation of a variety of substrates by hydrogen peroxide (H_2_O_2_). The accepted catalytic mechanism, a two-electron oxidation of native LPO-[Fe^III^] (Figure 1A, Equation (1)) followed by O-atom transfer from the enzyme to the substrate (Figure 1A, Equation (2)), is often referred to as the “halogen cycle”. The intermediate enzyme species, Compound I, is an oxoiron (IV) radical, with the organic radical located on the porphyrin (Cpd I-[Fe^IV^=O, π^+**.**^]) [2]. According to this widely accepted mechanism, substrate selectivity is determined by the rate of the reaction of Cpd I with potential substrates [3], which in turn is a function of the rate constants for the second step of the halogen cycle mechanism and the concentrations of the substrates in various physiologic fluids [4]. Although iodide (I^−^) reacts with LPO with a significant rate constant, the pseudo-halide thiocyanate (SCN^−^) is generally more abundant in physiologic fluids, and it is believed to be the exclusive substrate of LPO in vivo. Thus, the antimicrobial activity of the LPO system is attributed to hypothiocyanite (OSCN^−^) [5].

When the reaction of LPO-[Fe^III^] with H_2_O_2_ is rate-limiting, as has been reported for SCN^−^ [3], the kinetics of the halogen cycle mechanism are expected to be first-order in [H_2_O_2_] and independent of reducing substrate concentration ([X^−^]). This is what is observed at high concentration of X = SCN^−^ (relative to the concentration of H_2_O_2_). However, we report here that the catalytic reaction becomes biphasic at a low concentration of reducing substrate (relative to H_2_O_2_), a first-order reaction followed by a zeroth-order reaction. A new mechanism is proposed that accounts for the kinetics under all reaction conditions—a reverse-ordered sequential mechanism where the initial reaction is the binding of LPO-[Fe^III^] with SCN^−^ (Figure 1B, Equation (3)), not H_2_O_2_, as described by the halogen cycle. The resulting bound LPO species (LPO-SCN) subsequently reacts with H_2_O_2_ irreversibly in the turnover-limiting step to produce native LPO-[Fe^III^] and hypothiocyanite (OSCN^−^) (Figure 1B, Equation (4)). However, H_2_O_2_, in addition to acting as the oxidizing agent, also acts as a tight binding inhibitor of LPO-[Fe^III^] to produce a LPO-H_2_O_2_ bound species which is inactive or less active in catalysis (Figure 1B, Equation (5)). Through time-resolved spectral deconvolution, we have identified the LPO-H_2_O_2_ bound species as Compound I* (Cpd I*-[Fe^IV^-OH, aa^+**.**^], an oxoiron(IV) radical like Cpd I, albeit protonated [6,7,8], but with the organic radical relocated from the porphyrin to an amino acid). The possible biological significance of the new mechanism is discussed.

## 2. Materials and Methods

### 2.1. Materials

Water was doubly distilled in glass. The concentrations of H_2_O_2_ (ε_240nm_ = 36.4 M^−1^cm^−1^), LPO (ε_412nm_ = 112,000 M^−1^cm^−1^), and TNB (5-thio-2-nitrobenzoic acid, ε_412nm_ = 14,150 M^−1^cm^−1^) were determined spectrophotometrically. Solutions of NaSCN and LPO were buffered in 100 mM pH 7 phosphate (prepared using NaH_2_PO_4_, Na_2_HPO_4_, and NaOH). Buffers were treated with Chelex-100.

### 2.2. Instruments

pH was measured using an Orion Research Expandable ionAnalyzer EA 920. Absorbance measurements were made with an HP 8452A diode array spectrophotometer. Stopped-flow data were collected using a Hi-Tech SF-661 DX2 instrument (TgK Scientific Limited, Bradford-on-Avon, United Kingdom) equipped with a xenon arc lamp, a 1.00 cm path length quartz sample cell, and a photomultiplier tube for monochromatic detection or a diode array for collection of polychromatic data.

### 2.3. Stopped-Flow Data

Single mixing stopped-flow experiments according to the scheme in Figure S1 were used to probe the mechanism of the LPO-catalyzed oxidation of SCN^−^ and H_2_O_2_. Experiments were done under what will be considered “high concentration” and “low concentration” of reducing substrates. High concentration of reducing substrates refers, in general, to experiments we conducted where [SCN^−^] ≥ 1 mM, [LPO] ≥ 1 µM, and pseudo-first-order conditions of [SCN^−^] with respect to [H_2_O_2_] were used. Low concentration of reducing substrates refers, in general, to experiments conducted under conditions of [SCN^−^] ≤ 1 mM, stoichiometric or near stoichiometric conditions of [SCN^−^] with respect to [H_2_O_2_], and [LPO] ≤ 1 µM. High and low concentration conditions were optimized to observe the effects of varying reactant and enzyme concentrations where first-order and biphasic reaction trace kinetics were observed, respectively. Experimental traces were collected at 412 nm to observe the change in TNB during the LPO catalysis. Five runs were averaged and fit to a Mathematica model described in the Supplementary Materials. Data from the experiments where the biphasic reaction kinetics were observed were collected for the pre-steady-state reaction in-dependently to obtain more resolved traces. Data were then collected over the entire reaction time and the data sets were merged.

### 2.4. Effect of Mixing Order

The effect of the order of mixing was determined under low [SCN-] conditions. Two double mixing stopped-flow experiments were performed which first allowed LPO and SCN^−^ to come to equilibrium in the first mixing cycle, then added H_2_O_2_ in the second mixing cycle to initiate catalysis. A second double mixing experiment reacted LPO with H_2_O2 in the first mixing cycle, then added SCN^−^ in the second mixing cycle. The ageing time in both experiments was 1 s. Schematics of the mixing schemes are summarized in Figure S2 (for data of Figure 3A) and Figure S3 (for data of Figure 3B).

### 2.5. Data Analysis

Monochromatic kinetic data was analyzed using Kinetic Studio Version 1.0.12.19577. Poly-chromatic kinetic data were deconvoluted and fit using SPECFIT Version 3.0.40. Least-squares analysis to determine the relationship between observed rate constants was carried out using KaleidaGraph Version 3.5. Mathematica was used to model the kinetic data and to perform nonlinear fits to determine the rate constants. The code for the Mathematica model and additional details regarding data analysis are available in the Supplementary Text.

## 3. Results and Discussion

While the results and discussions are comingled, we have carefully separated our observations from interpretation. For example, kinetics and the rate law only suggest the chemical composition of the species involved, and not their structures. Accordingly, after a model is presented that describes the kinetics, we discuss the possible structures of species in the context of extra-kinetic data and the literature. Note the rate law provides insight into the turnover-limiting step of the catalytic cycle and the reversible steps that precede it; however, kinetics are silent in the subsequent steps of the catalytic cycle. Specifically, kinetics do not speak to the chemical nature of the species formed when LPO-SCN reacts with H_2_O_2_.

### 3.1. General Observations of Kinetics at High [SCN^−^]

When [SCN^−^] ≥ 1 mM, [LPO] ≥ 1 µM, and pseudo-first-order conditions of [SCN^−^] with respect to [H_2_O_2_] are used, exponential kinetics are observed with first-order dependence on the [H_2_O_2_] (Table S1) and first-order dependence on [LPO]. Slight systematic dependence on the [SCN^−^] is observed (Table S2). Accordingly, under conditions of high [SCN^−^] the experimental data essentially reflect the order dependencies predicted by the LPO halogen cycle model when the first step is rate-limiting (Figure 1A, Equation (1)).

### 3.2. General Observations of Kinetics at Low [SCN^−^]

**Figure 2:**
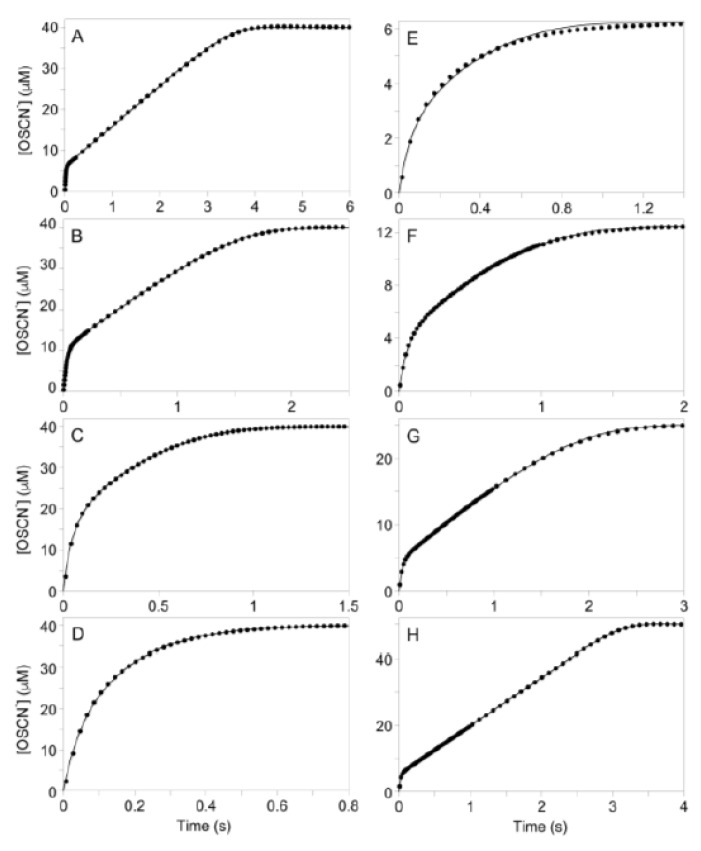
Experimental data (10% shown) and least-squares fit to the model of Figure 1B of the time traces for the LPO-catalyzed oxidation of SCN^−^ by H_2_O_2_ under conditions of low [SCN^−^]. (**A**–**D**): Effect of varying [SCN^−^]. Post-mixing concentrations were [LPO] = 1.2 µM, [H_2_O_2_] = 40 µM, [TNB] = 100 µM, and [SCN^−^] = 52, 106, 213, and 425 µM for A, B, C, and D, respectively. (**E**–**H**): Effect of varying [H_2_O_2_]. Post-mixing concentrations were [LPO] = 0.5 µM, [SCN^−^] = 100 µM, [TNB] = 100 µM, and [H_2_O_2_] = 6.25, 12.5, 25, and 50 µM for E, F, G, and H, respectively. For E-H, note the change in ordinate scale due to varying [H_2_O_2_]. Rate constants produced by these fits are given in Tables S6 and S7. Rate constants produced when [LPO] is varied (data not shown) are given in Table S8.

The mechanism of Figure 1A does not predict any change in the kinetics under conditions of low [SCN^−^], when k_2_ > k_1_, unless [H_2_O_2_] greatly exceeds [SCN^−^]. However, we observe biphasic kinetics with a first-order reaction followed by a zeroth-order reaction when [SCN^−^] < 1 mM, with stoichiometric or near stoichiometric [SCN^−^] with respect to [H_2_O_2_], and [LPO] ≤ 1 µM (e.g., Figure 2). Importantly, the first phase of the reaction is not a classic “burst phase”, as more than one enzyme turn-over is involved. Additionally, under the conditions of our experiments, the [SCN^−^] remains constant throughout the reaction because of the stoichiometry of the assay (TNB is 2-nitro-5-thiobenzoic acid and DTNB is the corresponding disulfide):



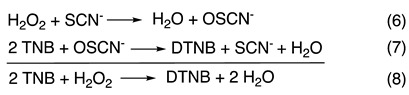



All of the experiments (except those that produce Figure 5) were carried out in the presence of TNB.

### 3.3. Kinetics at Low [SCN^−^] as a Function of [SCN^−^]

When the [SCN^−^] is relatively low, biphasic kinetics are observed with a first-order reaction followed by a zeroth-order reaction (Figure 2). However, as the [SCN^−^] is increased, the number of enzyme turnovers during the first phase of the reaction also increases (Figure S4). When the [SCN^−^] becomes sufficiently high, the kinetics become strictly first-order and the reaction rate became largely independent of [SCN^−^] (Figure 2D). When the pre-steady-state reaction is fit to a first-order model, the observed rate constant decreased with increasing [SCN^−^] (Figure S5). However, the dependence of the observed rate constant (k_obs_) on [SCN^−^] is not linear until at sufficiently high [SCN^−^], when the rate of the reaction becomes independent of [SCN^−^]. It was first hypothesized that SCN^−^ acts as an enzyme inhibitor; however, examination of initial reaction rates indicates that the reaction is independent of [SCN^−^] (Table S3). Instead, it appears that this initial reaction is a result of a pre-steady-state reaction, which is separate from the SCN^−^ oxidation catalysis and is inhibited by SCN^−^.

In contrast to the pre-steady-state reaction, the number of enzyme turnovers which occur during the (steady-state) zeroth-order reaction decrease as [SCN^−^] is increased. The reason for the decrease in the number of enzyme turnovers that occur during the steady-state reaction is two-fold. First, as H_2_O_2_ is the limiting reagent, the ratio of H_2_O_2_ reacted in the pre-steady-state versus the steady-state reaction increases until the steady-state reaction is no longer observed. Second, the rate of the steady-state reaction exhibits first-order dependency on [SCN^−^] (Figure S6). This causes the two reactions to become less resolved and more difficult to distinguish, particularly at higher [SCN^−^] (e.g., traces C and D of Figure 2).

### 3.4. Kinetics at Low [SCN^−^] as a Function of [H_2_O_2_]

Figure 2 Right shows the kinetic traces with varying [H_2_O_2_]. The first step of the biphasic reaction, which represents the pre-steady-state reaction, exhibits a first-order dependency on [H_2_O_2_] (Figure S7). The [H_2_O_2_] was limited to approximately 5–50 µM due to detection limits of the TNB spectrophotometric assay. The number of enzyme turnovers occurring during the pre-steady-state reaction increases only slightly with increasing [H_2_O_2_] and eventually, at sufficiently high [H_2_O_2_], the number of enzyme turnovers that occur during the pre-steady-state reaction remains constant (Figure S8).

The rate of the steady-state reaction is largely independent of [H_2_O_2_] (Table S4). The slight variation in the observed rate constants for the steady-state reaction at low [H_2_O_2_] is due to inaccuracies in modeling the decreasing linear phase of the reaction (e.g., traces F vs. G in Figure 2), which is reflected in an increased estimated error of the individual fits as [H_2_O_2_] is decreased (Table S4). The independence of the rate of the steady-state reaction on [H_2_O_2_] indicates either H_2_O_2_ is not directly involved in the reaction represented by the steady-state kinetics or the rate of enzyme turnover governs the rate of this reaction and steady-state active enzyme concentration is independent of [H_2_O_2_].

### 3.5. Kinetics at Low [SCN^−^] as a Function of [LPO]

The kinetics of the steady-state reaction act predictably as [LPO] is varied for a catalysis reaction with a first-order dependence on [LPO] (Figure S9). However, when the data are fit to a biphasic kinetic model, the observed rate constant of the pre-steady-state reaction is independent of [LPO] (Table S5). Additionally, the number of turnovers that occur during the pre-steady-state reaction increases with increasing [LPO] (Figure S10). This indicates that increasing [LPO] increases the rate of turnover but has no effect on the observed rate constant of the inhibitory reaction which results in the pre-steady-state reaction (Figure S10). This would occur if the inhibitory reaction were not catalytic in nature, but rather pseudo-first-order in SCN^−^ or H_2_O_2_ with respect to LPO (cf. the aforementioned dependencies of the pre-steady-state kinetics on [SCN^−^] and [H_2_O_2_]).

### 3.6. Reversible Binding of H_2_O_2_

**Figure 3:**
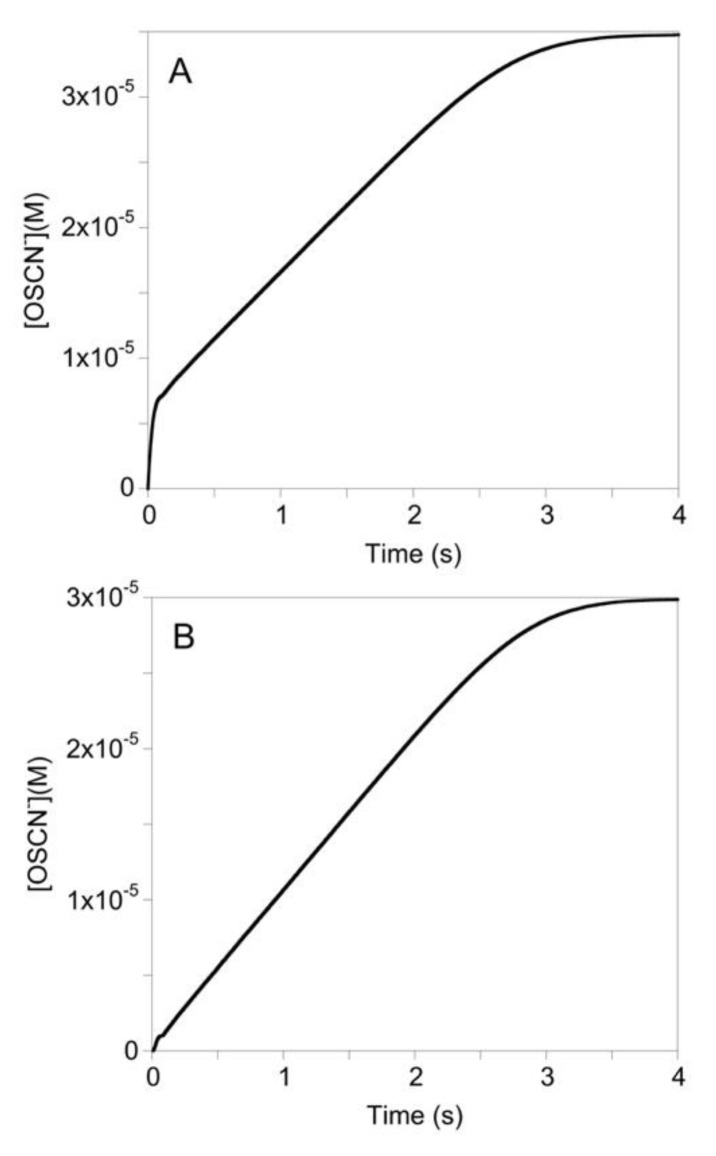
Kinetic traces of the LPO-catalyzed oxidation of SCN^−^ by H_2_O_2_ under conditions of low concentration of [SCN^−^] with (**A**) and without (**B**) LPO/SCN^−^ pre-equilibrium prior to mixing with H_2_O_2_. For A and B: the post-mixing concentrations were [LPO] = 1 µM, SCN^−^] = 100 µM, [H_2_O_2_] = 40 µM, and [TNB] = 100 µM. For B, the age time was 1 s.

In addition to concentration-dependence studies, the effect the order of mixing of LPO, SCN^−^, and H_2_O_2_ has on the reaction kinetics was also investigated. Most experiments conducted to observe the LPO-catalyzed oxidation of SCN^−^ by H_2_O_2_, LPO and SCN^−^ were mixed by hand, then this solution was mixed with H_2_O_2_ in a single mixing stopped-flow experiment. The reactions observed when this mixing order was used were biphasic as seen in Figure 3A. This mixing order was used to protect the enzyme from permanent inactivation by H_2_O_2_, as described in the literature [9,10,11,12,13]. However, to test the hypothesis that the reaction of LPO with H_2_O_2_ reversibly produces an inactive form of the enzyme, H_2_O_2_ was reacted with LPO to produce the inactive species in the first mixing step of a double mixing stopped-flow experiment. After an age time of one second (time to establish preequilibrium), SCN^−^ was mixed with the LPO-H_2_O_2_ species. When LPO was reacted with H_2_O_2_ prior to mixing with SCN^−^, the pre-equilibrium could no longer be observed, but the same kinetics for the zero-order phase are observed, indicating that an inactive enzyme species was reversibly produced by the reaction of LPO with H_2_O_2_ in the first mixing cycle (Figure 3B).

### 3.7. Proposed Kinetic Mechanism

The literature mechanism of Figure 1A does not describe all the kinetics that we have reported herein, and specifically the kinetics observed for the LPO-catalyzed oxidation of SCN^−^ by H_2_O_2_ at low [SCN^−^] (Figure 2). The model of Figure 1B depicts a reverse-ordered (SCN^−^ before H_2_O_2_) sequential mechanism with tight binding inhibition by H_2_O_2_. The first productive step of the reaction is the reversible binding of LPO-[Fe^III^] and SCN^−^ to produce LPO-SCN. This bound species then reacts irreversibly in the turnover-limiting step with H_2_O_2_ to produce native LPO-[Fe^III^] and OSCN^−^. In addition, H_2_O_2_ acts as a tight binding inhibitor by reacting reversibly with native LPO-[Fe^III^]. The structure of the LPO-H_2_O_2_ species will be discussed later.

### 3.8. Modeling of the Proposed Mechanism

A mathematical model was constructed for the mechanism of Figure 1B (Supporting Information). To account for the TNB assay (Equation (8)), the model assigns [SCN^−^] to a constant. For the model to reflect the biphasic kinetics observed experimentally, the following conditions must be met: (1) the binding equilibrium for LPO-[Fe^III^] and X^−^ must not be rate-limiting and lie toward the bound species, (k_3_ > k_4_) and (k_3_ >> k_−3_); (2) the reaction of LPO-[Fe^III^] with H_2_O_2_ to form the unproductive LPO-H_2_O_2_ species must be competitive with the reaction of LPO-SCN with H_2_O_2_ (k_4_ ≈ k_5_); and (3) the equilibrium which produces the unproductive LPO-H_2_O_2_ species must lie toward the H_2_O_2_-bound species (k_5_ >> k_−5_). Under these three conditions, the proposed mechanism qualitatively describes what is observed experimentally.

**Figure 4:**
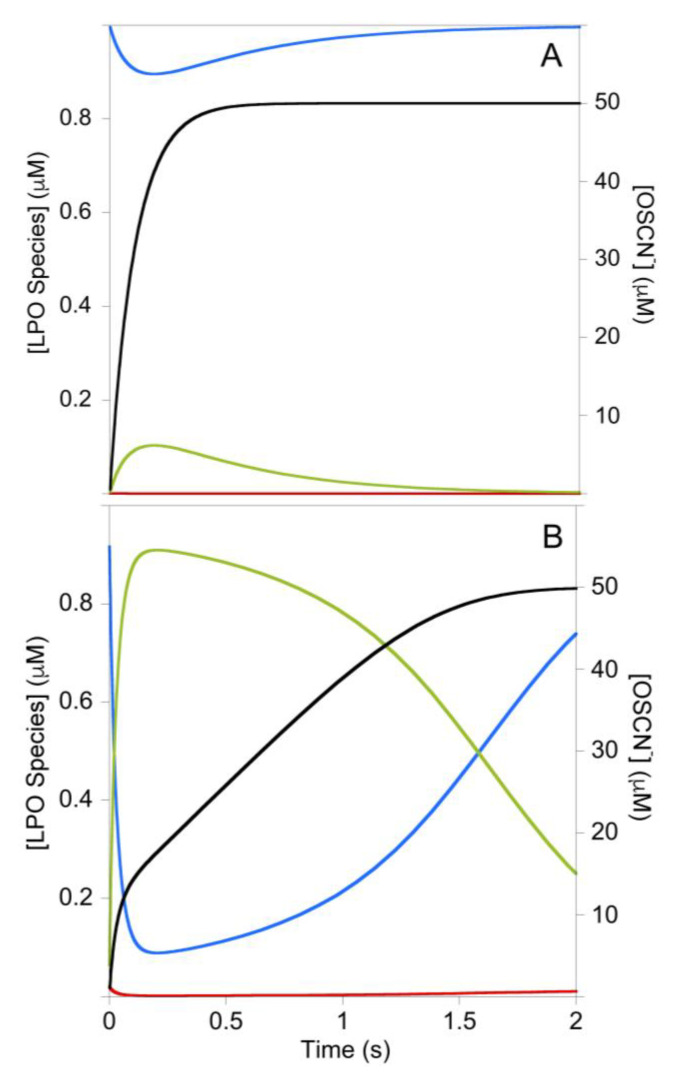
Simulated concentrations during the LPO-catalyzed oxidation of SCN^−^ by H_2_O_2_ for the mechanism of Figure 1B under high (**A**) and low (**B**) [SCN^−^] conditions: Red = LPO-[Fe^III^], Blue = LPO-SCN, Green = Cpd I*, Black = OSCN^−^. For A and B: k_3_ = 1.4 × 10^9^ M^−1^s^−1^, k_−3_ = 1000 s^−1^; k_4_ = 1 × 10^7^ M^−1^s^−1^; k_5_ = 3.48 × 10^7^ M^−1^s^−1^, k_−5_ = 2.07 s^−1^; [LPO]_0_ = 1 µM; [H_2_O_2_]_0_ = 50 µM. For A: [SCN^−^]_0_ = 1 mM. For B: [SCN^−^]_0_ = 50 µM. The conditions of Figure 4B are approximately the same as Figure 2A. This simulation assumes the [SCN^−^] remains constant, as expected for the stoichiometry of the TNB assay. The results of Figure 4B may be compared with a simulation of the mechanism of Figure 1B that included the known rate of conversion of Cpd I to Cpd I* (Figure S15), which predicts LPO-[Fe^III^] is the steady-state species and the reaction is pseudo-first-order.

The concentration of the enzyme species predicted by the mathematical model during the pre-steady-state and steady-state phases of the reaction under various [SCN^−^] provides insight into the kinetics. Under conditions of high [SCN^−^], LPO-SCN is produced rapidly and remains the primary enzyme species throughout the reaction (Figure 4A). As a result, the reaction observed under conditions of high [SCN^−^] is first-order with the turnover-limiting step being the reaction of LPO-SCN with H_2_O_2_ (Figure S11). When [SCN^−^] is low, the rapid equilibrium binding of native LPO-[Fe^III^] by SCN^−^ causes the fast production of the active LPO-SCN species. The competitive rate of k_4_ and k_5_ as well as the tight binding of H_2_O_2_ to native LPO-[Fe^III^] causes a relatively slow conversion from LPO-SCN as the primary enzyme species to the unproductive species LPO-H_2_O_2_ (Figure 4B). As LPO-SCN is the productive enzyme species in the turnover limiting step, the unproductive species LPO-H_2_O_2_ causes a decrease in the rate of OSCN^−^ production (Figure S12). After steady-state concentrations have been reached, the reaction becomes zeroth-order (Figure S13). During the steady-state reaction, the concentration of the unproductive enzyme species, LPO-H_2_O_2_, slowly decreases and is replaced by the SCN^−^ bound species, LPO-SCN. Thereafter, until H_2_O_2_ has been depleted and the reaction is complete, LPO-SCN remains the primary enzyme species (Figure 4B).

### 3.9. Fitting the Experimental Data

The aforementioned Mathematica model was used to fit experimental data and to calculate the rate constants. The data for which the pre-steady-state reactions were well resolved from the steady-state reactions, as in Figure 2A,B, were easily fit by the model and all the rate constants were fit simultaneously. However, the rate constants for the data in which the pre-steady-state and steady-state reactions were not well resolved, as in Figure 2C, could not be fit simultaneously. In these cases, rate constants which were not primary to the observed kinetics were fixed so the rate constants related to the reactions which were dominant could be calculated (Tables S6–S8). Representative fits of the kinetic data when the [SCN^−^] and [H_2_O_2_] were varied are illustrated in Figure 2. Representative fits of the kinetic data when he [LPO] was varied are illustrated in Figure S14. Table 1 summarizes the average calculated rate constants for the model proposed in Figure 1B. The predictions made concerning the relative magnitudes of the rate constants appear to be correct, i.e., (k_3_ > k_4_), (k_3_ >> k_−3_), (k_4_ ≈ k_5_), and (k_5_ >> k_−5_). The rate constant k_3_ is near diffusion-controlled.

### 3.10. The Structure of LPO-H_2_O_2_

**Figure 5:**
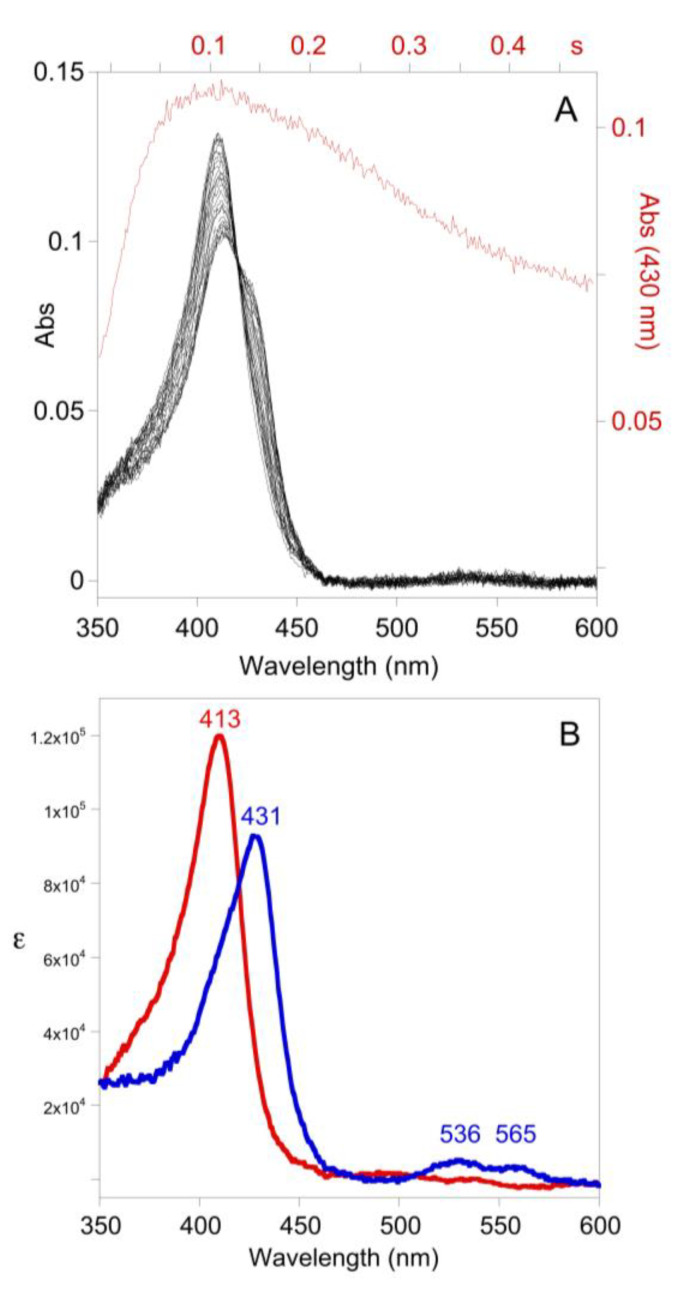
(**A**) Time-resolved UV-visible spectra (10% of data shown) and trace at 430 nm (cf. the green trace of Figure 4B). Post-mixing concentrations were [LPO] = 1.2 µM, [H_2_O_2_] = 40 µM, and [SCN^−^] = 52 µM at pH 7. (**B**) Deconvoluted color components from singular value decomposition of the time-resolved spectra of A, LPO-[Fe^III^]/LPO-SCN (red) and Cpd I*-[Fe^IV^-OH, aa^+.^] (blue).

The simulation of Figure 4B predicts the intermediate species LPO-H_2_O_2_ should accumulate during catalysis in the presence of low [SCN^−^]. Time-resolved spectra were collected (in the absence of TNB) and subjected to singular value decomposition analysis. Model-free analysis predicted two colored species, but it was subsequently determined that the model of Figure 1B required the electronic spectrum of LPO-[Fe^III^] to be similar to LPO-SCN. In reality, binding of SCN^−^ to LPO results in a pH-dependent red shift of 1–4 nm [14], which is not resolved by the rapid-scan diode-array of the stopped-flow instrument. It is not surprising that the spectra of LPO and LPO-SCN are similar as SCN^−^ is known to bind remotely relative to the iron-heme chromophore [15]. Subsequent singular value decomposition analysis yields a spectrum similar to LPO-[Fe^III^], albeit red-shifted by about 1 nm (which we interpret is actually LPO-SCN, cf. Figure 4B), and a spectrum that appears to be Compound II (Cpd II-[Fe^IV^-OH], without a radical on the porphyrin or amino acid backbone). Cpd II-like species are known to form spontaneously from Cpd I [2]. Electronic absorption spectra do not distinguish between true Cpd II species, which are the result of one-electron reduction of Cpd I, and species like Cpd I-[Fe^IV^-OH, aa^+.^], where the radical has moved from the porphyrin to an amino acid, but EPR can distinguish these species [16]. Since the LPO-H_2_O_2_ species forms reversibly in the absence of one-electron donors (Figure 5A) and the deconvoluted spectrum is identical to Cpd I*-[Fe^IV^-OH, aa^+.^] (Figure 5B, in addition to the Soret band at 431, the Q-bands at 536 and 565 are diagnostic) [3,17], we conclude the latter is LPO-H_2_O_2_. We note there is kinetic and spectroscopic evidence that protein radicals are formed during LPO turnover [16,18]. While the electronic spectra of Cpd I*-[Fe^IV^-OH, aa^+.^] and Cpd II-[Fe^IV^-OH] are similar, we ruled out Cpd II-[Fe^IV^=O] for LPO-H_2_O_2_ because it is known to be stable for more than five minutes [19] and because the formation of LPO-H_2_O_2_ is reversible (cf. Figure 3 and Figure 5A). Importantly, a clean isosbestic point exists at 420 nm for the conversion of LPO-[Fe^III^] to Cpd I*-[Fe^IV^-OH, aa^+.^] during catalysis (Figure 5). In contrast, the isomerization of Cpd I-[Fe^IV^=O, π^+.^] to Cpd I*-[Fe^IV^-OH, aa^+.^] occurs with a rate constant of 2 s^−1^ [3] and the reaction has an isosbestic point at 408 nm [18]. The presence of the isosbestic point at 420 nm in Figure 5 and a simulation (Figure S15) show that the production of Cpd I*-[Fe^IV^-OH, aa^+.^] during catalysis either does not involve Cpd I-[Fe^IV^=O, π^+^.], or if it does, the conversion of Cpd I-[Fe^IV^=O, π^+.^] to Cpd I*-[Fe^IV^-OH, aa^+.^] has been accelerated. In our model, LPO-H_2_O_2_ does not react directly with SCN^−^, which implies Cpd I-[Fe^IV^=O, π^+.^] is not captured by SCN^−^ during catalysis.

### 3.11. Proposed Intimate Mechanism

**Figure 6:**
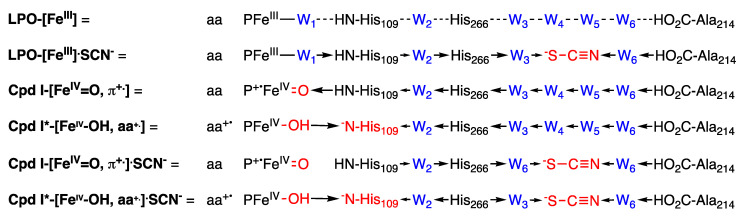
Proposed intimate mechanism for the LPO-catalyzed oxidation of thiocyanate and inhibition by hydrogen peroxide. Significant modifications to the resting state are indicated in red and the resulting changes in H-bond donation are indicated by arrows. **LPO-[Fe^III^]** is stabilized by an aqua ligand (W_1_) that is connected to a H-bonded chain the extends the length of the substrate diffusion channel. The binding of SCN^−^ displaces two waters in the chain (**LPO-[Fe^III^]^.^SCN^−^**), whereupon H-bond donation is reorganized to stabilize the negative charge on SCN^−^. The oxo ligand (O^2−^) is stabilized by the H-bond change in **Cpd I-[Fe^IV^=O, π^+.^]**. Proton transfer to Cpd I coupled with electron donation by an amino acid yield Cpd I*-[Fe^IV^-OH, aa^+.^], where His_109_ anion is stabilized by the H-bond chain. The binding of SCN^−^ to Cpd I results reorganizes the H-bond chain **Cpd I-[Fe^IV^=O, π^+.^]^.^SCN^−^** to stabilize SCN^−^ while destabilizing the [Fe^IV^=O] moiety. The binding of SCN^−^ to Cpd I* results in conflict in the H-bond chain of Cpd I*-[Fe^IV^-OH, aa^+.^]^.^SCN^−^.

While the kinetic mechanism of Figure 1B is consistent with the experimental results that are presented herein, it does not identify the species formed when LPO-SCN reacts with H_2_O_2_, nor does it offer insight into why the sequence of substrate addition is important. Regarding the species formed after the turnover-limiting step of catalysis, it is likely a Compound I-like species, albeit bound by SCN^−^, although none of our results address its structure. Regarding the sequence of substrate addition, the intimate mechanism must involve an interplay of redox potentials, acid/base properties, and activation barriers that are influenced by molecular geometrics. We offer the following interpretation that is based upon several extrakinetic observations. The distal heme cavity is connected to the surface by a 22 Å-long substrate diffusion channel that is lined by hydrophobic residues [20]. A hydrogen bonded chain involving Fe^3+^-W_1_-His_109_-W_2_-His_266_-W_3_-W_4_-W_5_-W_6_-Ala_214_ within the substrate diffusion channel connects the heme to the surface, where His_109_ is believed to be a key proton donor at the heme and waters W_1–6_ are generally conserved [20]. The pK_a_ of HSCN is −1.4 (so it is an anion that must be charge-neutralized in the binding pocket). SCN^−^ binds in the distal heme cavity by displacing two of the conserved waters, thereby disrupting the hydrogen bond chain [20]. Finally, Cpd I*-[Fe^IV^-OH, aa^+.^] is protonated at the terminal oxo ligand (which is curious as Cpd I-[Fe^IV^=O, π^+.^] is not) [6,7,8]. Figure 6 summarizes the potential LPO species that are involved in catalysis. The turn-over limiting step (Figure 1B, Equation (4)) involves a rate-limiting reaction of H_2_O_2_ with LPO-[Fe^III^]^.^SCN^−^ to presumably give Cpd I-[Fe^IV^=O, π^+.^]^.^SCN^−^. Histidines that are buried in proteins (pK_a_ = ~7 ± 1) tend to be less acidic than free histidine (pK_a_ = ~6) [21]. The large variation of His pK_a_ in proteins reflects intrinsic differences due to the specific environment of each residue. The optimal pH for the LPO system is ~6, although the optimal value depends on the particular substrate [22]. It is likely the variability is due to the local environments of His_109_ and/or His_266_ (as well as other residues). We propose the proximity of the anionic SCN^−^ substrate combined with the disruption of the hydrogen bond chain increases the pK_a_ of His_109_, preventing it from stabilizing the oxo ligand of Cpd I-[Fe^IV^=O, π^+.^]^.^SCN^−^ (Figure 6) and facilitating the reaction of LPO-[Fe^III^]^.^SCN^−^ with H_2_O_2_ (Figure 1B, Equation (4)). In contrast, in the absence of SCN^−^, His_109_ is capable of protonating the oxo ligand of Cpd I-[Fe^IV^=O, π^+.^], thereby converting it to Cpd I*-[Fe^IV^-OH, aa^+.^] by facilitating electron transfer. The intimate model of Figure 6 furthermore suggests reasons why LPO-[Fe^III^] binds SCN^−^, whereas, due to conflict in H-bonding, Cpd I*-[Fe^IV^-OH, aa^+.^] does not.

Biological relevance. The physiological relevance of the mechanism of Figure 1B may be traced to the role of LPO in health and disease. LPO is secreted by mucosal glands to body fluids including breast milk, salivary, lachrymal, and airway secretions. In general, health is associated with relatively high [SCN^−^] and low [H_2_O_2_]. However, disease states frequently reduce [SCN^−^] and increase [H_2_O_2_], and LPO activity is consequently impaired for diseases like caries [23], cystic fibrosis [24], and neonatal pathologies [25].

Low [SCN^−^] develops through two distinct mechanisms in vivo, defective transport and chemical reaction. Endocrine fluids are generally characterized by high [SCN^−^]. For example, active transport into airway secretions and saliva of non-smokers produce about 500 µM SCN^−^ (although mM concentrations are observed for smokers), which is orders of magnitude higher than plasma, which is typically ~35 µM [26,27]. In contrast, SCN^−^ release is absent in lung epithelial cells from mutations in the cystic fibrosis transmembrane conductance regulator (CFTR) [28,29]. Furthermore, xerostomia (dry mouth)—often a side effect of certain medications, aging issues, or as a result of radiation therapy for cancer—renders the principal source of SCN^−^ in the oral cavity ineffective. Even when transport is functional, low [SCN^−^] can result from a local chemical reaction, especially under conditions of oxidative stress that deplete thiols, the exclusive reactants of OSCN^−^. Without reaction partners, OSCN^−^ rapidly decomposes through irreversible processes, thereby yielding a pathway that chemically depletes SCN^−^ [30]. Both mechanisms can reduce SCN^−^ to concentrations comparable to Figure 4B.

High [H_2_O_2_] can be produced by the host and by certain infectious agents. Concentrations of H_2_O_2_ in excess of 100 µM in human physiological fluids are not uncommon [31]. Some human pathogens, such as mutans streptococci (including cariogenic *Streptococcus mutans* and *S. sobrinus*), accumulate up to 2 mM H_2_O_2_ in their media during growth on glucose [32]. While H_2_O_2_ is present in the air exhaled by healthy human subjects, amounts of exhaled H_2_O_2_ appear greater in subjects with inflammatory lung diseases and in cigarette smokers [31].

At sufficient concentrations, H_2_O_2_ is cytotoxic to mammalian cell lines, including human epithelial cells [33] and gingival fibroblasts [34,35]. In the presence of SCN^−^, the LPO system protects cultured mammalian cells against H_2_O_2_ toxicity [36]. This is consistent with the observation that OSCN^−^ is not toxic toward mammalian cells [37,38]. It has been previously suggested that one of the important roles of human peroxidases is to detoxify H_2_O_2_ to prevent host tissue damage [39,40]. It is generally assumed that detoxification occurs during the consumption of H_2_O_2_ to produce OSCN^−^. However, that process is retarded when the [H_2_O_2_] is high and the [SCN^−^] is low (cf. Figure 4A vs. Figure 4B), conditions that are favored in disease and that promote the accumulation of Cpd I*-[Fe^IV^-OH, aa^+.^]. Furthermore, LPO is an ineffectual catalase [41]. Accordingly, not only must the LPO mechanism be revised, but the physiological behavior of LPO in disease requires further consideration in the context of our findings. In addition, the mechanisms of other members of the human peroxidase family, especially myeloperoxidase and eosinophil peroxidase, should be reexamined.

## 4. Conclusions

To account for new data that are inconsistent with the previously accepted mechanism for the LPO-catalyzed oxidation of SCN^−^ by H_2_O_2_, an ordered sequential mechanism is proposed herein where the order of substrate binding is reversed, first SCN^−^ and then H_2_O_2_. In the new model, the sequence of substrate binding that is described by the literature halogen cycle mechanism is inhibitory. As the reaction conditions that give rise to the proposed catalytic mechanism may be biologically relevant, the role of LPO in human physiology deserves further consideration.

## Figures and Tables

**Table 1:** Apparent Rate Constants for the Mechanisms of Figure 1. Estimated error of the least-significant figure given in parentheses.

Constant	Value	Temp (°C)	Source
k_1_	1.1 × 10^7^ M^−1^s^−1^	15	3
k_2_	2.0 × 10^8^ M^−1^s^−1^	15	3
k_3_	1.4(2) × 10^9^ M^−1^s^−1^	25	this study
k_−3_	3.94(5) × 10^3^ s^−1^	25	this study
k_4_	1.0 (3) × 10^7^ M^−1^s^−1^	25	this study
k_5_	3(1) × 10^7^ M^−1^s^−1^	25	this study
k_−5_	1.9(4) s^−1^	25	this study

## Data Availability

The data described in this study are available [42].

## Supplementary Materials

The following are available online at https://www.mdpi.com/article/10.3390/antiox10111646/s1, a discussion of related literature for myeloperoxidase (MPO), Mathematica code for fitting and modeling the data, Figure S1: Reaction mixing scheme for the single mixing stopped-flow experiment to observe the rate of the LPO-catalyzed oxidation of SCN^−^ by H_2_O_2_ at pH 7., Figure S2: Reaction mixing scheme for the double mixing stopped-flow experiment to observe the effect of mixing LPO and SCN^−^ in the first mixing cycle prior to the addition of H_2_O_2_ on the LPO-catalyzed oxidation of SCN^−^ by H_2_O_2_ at neutral pH, Figure S3: Reaction mixing scheme for the double mixing stopped-flow experiment to observe the effect of mixing LPO and H_2_O_2_ in the first mixing cycle prior to the addition of SCN^−^ on the LPO-catalyzed oxidation of SCN^−^ by H_2_O_2_ at neutral pH, Figure S4: Isolated view of the pre-steady-state reaction observed in Figure 2 (left) for the LPO-catalyzed oxidation of [SCN^−^] by H_2_O_2_ under low [SCN^−^] conditions, Figure S5: Observed first-order rate constants for the pre-steady-state reaction in the LPO-catalyzed oxidation of SCN^−^ by H_2_O_2_., Figure S6: Effect of varying [SCN^−^] on the rate of the steady-state reaction during the LPO-catalyzed oxidation of SCN^−^ by H_2_O_2_, Figure S7: Effect of [H_2_O_2_] on k_1obs_ for the LPO-catalyzed oxidation of SCN^−^ by H_2_O_2_ under conditions of low concentration of [SCN^−^], Figure S8: Kinetic traces of the pre-steady-state reaction observed during the LPO-catalyzed oxidation of SCN^−^ by H_2_O_2_ as [H_2_O_2_] was varied, Figure S9: Steady-state reaction rate dependence on [LPO] for the LPO-catalyzed oxidation of SCN^−^ by H_2_O_2_, Figure S10: Kinetic traces of pre-steady-state reaction of the LPO-catalyzed oxidation of SCN^−^ by H_2_O_2_ as [LPO] is varied, Figure S11: Simulated trace of [OSCN^−^] for the proposed mechanism for the LPO-catalyzed oxidation of SCN^−^ by H_2_O_2_ under conditions of high [SCN^−^], Figure S12: Simulated trace of [OSCN^−^] during the pre-steady-state reaction for the proposed mechanism for the LPO-catalyzed oxidation of SCN^−^ by H_2_O_2_ under conditions of low [SCN^−^], Figure S13: Simulated trace of [OSCN^−^] during the reaction for the proposed mechanism for the LPO-catalyzed oxidation of SCN^−^ by H_2_O_2_ under conditions of low [SCN^−^], Figure S14: Fitted kinetic traces for the LPO-catalyzed oxidation of SCN^−^ by H_2_O_2_ as [LPO] was varied, Figure S15: Simulated concentrations during the LPO-catalyzed oxidation of SCN^−^ by H_2_O_2_ for the mechanism of Figure 1A with the additional known step for the conversion of Cpd I to Cpd I*, Table S1: Observed rate of the LPO-catalyzed oxidation of SCN^−^ by H_2_O_2_ as a function of [H_2_O_2_], Table S2: Observed rate of the LPO-catalyzed oxidation of SCN^−^ by H_2_O_2_ as a function of [SCN^−^], Table S3: Observed initial rates of the LPO-catalyzed oxidation of SCN^−^ by H_2_O_2_ as a function of [SCN^−^], Table S4: Effect of varying [H_2_O_2_] on the rate of the steady-state reaction of the LPO-catalyzed oxidation of SCN^−^ by H_2_O_2_, Table S5: Effect of varying [LPO] on the rate of the pre-steady-state reaction for the LPO-catalyzed oxidation of SCN^−^ by H_2_O_2_, Table S6: Fitted rate constants for the proposed mechanism of the LPO-catalyzed oxidation of SCN^−^ by H_2_O_2_ as [SCN^−^] was varied, Table S7: Fitted rate constants for the proposed mechanism of the LPO-catalyzed oxidation of SCN^−^ by H_2_O_2_ as [H_2_O_2_] was varied, Table S8: Fitted rate constants for the proposed mechanism of the LPO-catalyzed oxidation of SCN^−^ by H_2_O_2_ as [LPO] was varied.
