# Supplementary material for: Reverse Ordered Sequential Mechanism for Lactoperoxidase with Inhibition by Hydrogen Peroxide
Source: Antioxidants (Basel). 2021 Oct 20;10(11):1646. doi: 10.3390/antiox10111646 (PMC8614691; doi:10.3390/antiox10111646)
Supplement: Supplementary file 1 [file antioxidants-10-01646-s001.zip › antioxidants-1353073-supplementary.pdf]

Reverse Ordered Sequential Mechanism for Lactoperoxidase with Inhibition by Hydrogen Peroxide

Kellye Cupp-Sutton and Michael T. Ashby*

Department of Chemistry and Biochemistry, University of Oklahoma, Norman, OK 73019

SUPPORTING MATERIALS

A Discussion of Related Literature for MPO. A Compound II-like species has been previously observed for MPO under conditions of low [SCN^-] (Schlorke, D.; Flemmig, J.; Gau, J.; Furtmüller, P. G.; Obinger, C.; Arnhold, J., "New Insights into thiocyanate oxidation by human myeloperoxidase," *J. Inorg. Biochem.*, **2016**, *162*, 117-126). The authors of the 2016 study offer a mechanism for MPO that is very different that the one that is described for LPO in the present work. However, there are several reasons a discussion of the 2016 study is problematic, including:

- The focus of the 2016 paper was on identifying transient MPO species. The work did not produce a rate law that explains their observations. Furthermore, no correlate was made between the observed MPO species and catalysis.

- Formation of Compound II-like species were observed in the presence of a 20,000-fold excess of $OSCN^-$ under conditions that are known to produce large amounts of reactive impurities (including cyanide, sulfite, cyanate, and cyanosulfite). Thus, it is impossible to attribute the reaction to $OSCN^-$.

- The products formed from the oxidation of SCN^- were characterized using ^{13}C NMR. This is not a quantitative method. In fact, the relaxation times for sp-hybridized ^{13}C are so long, it is impossible to observe complete relaxation of all of the unstable SCN-derived species during their lifetime.

- SVD analysis (cf. Figure 5) was not performed on the time-resolved spectral data. Thus, not only are the number of colored species unknown, but the values of λ_{max} are suspect.

- The 2016 study begins with the pre-formation of MPO Compound I, which we argue for LPO is never formed in significant concentrations. Furthermore, MPO Compound I was prepared using excess HOCl without consideration of the near diffusion-controlled reaction of HOCl with SCN^- and $OSCN^-$ in subsequent mixing cycles.

- We have previously shown (e.g., ref. 30) that the conditions that the authors employed in their 2016 paper to produce $OSCN^-$ under conditions of low [SCN^-] results in over-oxidation of SCN^- and the production of SCN^- species (including CN^-) that are not typically formed during catalysis. E.g., it is not possible to prepare 17 mM $OSCN^-$ by reacting HOCl with SCN^- without the production of large amounts of side products (cf. Figure 6 of the 2016 paper).

- In the 2016 paper, the rate of formation of MPO Compound II by the reaction of $OSCN^-$ with ferric MPO is reported to be $6x10^4$ $M^{-1}s^{-1}$. First, we do not believe this is actually the reaction of $OSCN^-$ with MPO, but rather the reaction of over-oxidation products. Also, we do not know what the corresponding rate constant would be for LPO. However, even if we assume the reaction is diffusion-controlled (10^9 $M^{-1}s^{-1}$), the reaction of ferric LPO with $OSCN^-$ (0.06 s^{-1}) would not be competitive with the reaction of $OSCN^-$ with TNB (90 s^{-1} for our Figure 3). Not only is there at least a 10^3 difference in the rate constants (more likely 10^9 difference if the rate constant is the same for LPO and MPO), but the reaction does not explain our results (e.g., Figure 2).

Modeling the Halogen Cycle Mechanism of LPO. Mathematica was used to model the LPO halogen cycle (Figure 1A). In the model EE = LPO, Ox = H_2O_2, EOx = Cpd I, S = SCN^-, P = $OSCN^-$. The rate constants are taken from Table 1.

```
Clear[EE, ES, EOx, S, Ox, P, Ox0, t, k1, k2];

equations = {
  EE'[t] == -k1*EE[t]*Ox[t] + k2*EOx[t]*S,
  EOx'[t] == +k1*EE[t]*Ox[t] - k2*EOx[t]*S,
  Ox'[t] == -k1*EE[t]*Ox[t],
  P'[t] == k2*EOx[t]*S,
  EE[0] == EE0,
  EOx[0] == 0,
  Ox[0] == Ox0,
  P[0] == 0
  };

k1 = 1.1*10^7;
k2 = 2*10^8;
EE0 = 1*10^-6;
S = 1*10^-4;
Ox0 = 5*10^-5;

timeframe = 5;

answer = NDSolve[equations, P[t] , {t, 0, timeframe}];

EE[t_] = EE[t] /. answer;
EOx[t_] = EOx[t] /. answer;
Ox[t_] = Ox[t] /. answer;
P[t_] = P[t] /. answer;
```

Modeling of the Proposed Mechanism of LPO. Mathematica was used to model the mechanism of Figure 1B. In the model EE = LPO, ES = LPO-SCN, Ox = H_2O_2, EOx = LPO-H_2O_2 = Cpd I*, S = SCN^-, and P = $OSCN^-$. The rate constants are taken from Table 1.

```
Clear[EE,ES,EOx,S, Ox, P,Ox0, t,k1, km1,k2, km2, k3];

equations = {
  EE'[t] == -k1*EE[t]*S+km1*ES[t]-k2*EE[t]*Ox[t]+km2*EOx[t]+k3*ES[t]*Ox[t],
  ES'[t] == k1*EE[t]*S-km1*ES[t]-k3*ES[t]*Ox[t],
  EOx'[t] == k2*EE[t]*Ox[t]-km2*EOx[t],
  Ox'[t] == -k3*ES[t]*Ox[t],
  P'[t] == k3*ES[t]*Ox[t],
  EE[0] == EE0,
  ES[0]== 0,
  EOx[0]== 0,
  Ox[0]== Ox0,
  P[0]== 0};

k1=1.4*10^9;
km1=3940;
k2=3.48*10^7;
km2=2.07;
k3=1*10^7;
EE0=1*10^-6;
S=1*10^-4;
Ox0=5*10^-5;

timeframe = 6;

answer=NDSolve[equations, {EE[t], ES[t], EOx[t],S[t], Ox[t], P[t]}, {t,0,timeframe}];

EE[t_]=EE[t]/.answer;
ES[t_]=ES[t]/.answer;
EOx[t_]=EOx[t]/.answer;
S[t_]=S[t]/.answer;
Ox[t_]=Ox[t]/.answer;
P[t_]=P[t]/.answer;
```

Fitting Experimental Data Using the Proposed Mechanism of Figure 1B. Experimental kinetic traces were fit to the model of Figure 1B at low [SCN^-] to determine rate constants k_1, k_{-1}, k_2, k_{-2}, and k_3. The reactions involving the TNB assay itself were omitted so as not to over complicate the model. The molar extinction coefficient, ε_{412}(TNB) = 14,150 $M^{-1}cm^{-1}$, was used to calculate [$OSCN^-$]. ParametricNDSolve was used to model the mechanism and NonlinearRegression was used to calculate fitted rate constants. Initial parameters were determined by allowing all rate constants to vary for a set of data where the pre-equilibrium and steady-state reaction could both be clearly observed, as in Figure 2B. The rate constants

calculated for well-resolved data were used as initial parameter values to fit the traces which were less well-defined. In some cases, where the pre-equilibrium reaction and steady-state reactions were especially underdetermined, such as traces C and D in Figure 2, appropriate rate constants were fixed to allow the calculation of the rate constants for the dominant reaction which could be clearly observed in the trace. In the model EE = LPO, ES = LPO-SCN, Ox = H_2O_2, EOx = LPO-H_2O_2 = Cpd I*, S = SCN^-, and P = $OSCN^-$.

```
SetDirectory["path"];
data1=ReadList["filename ",{Number,Number}];

Clear[EE,ES,EOx,S,Ox,P,k1,k2,k3,k4,k5];

totaltime=6;

S=5.3*10^-5;

model=ParametricNDSolveValue[
  {
  EE'[t] == -k1*EE[t]*S+k2*ES[t]-k3*EE[t]*Ox[t]+k4*EOx[t]+k5*ES[t]*Ox[t],
  ES'[t] == k1*EE[t]*S-k2*ES[t]-k5*ES[t]*Ox[t],
  EOx'[t] == k3*EE[t]*Ox[t]-k4*EOx[t],
  Ox'[t] == -k5*ES[t]*Ox[t],
  P'[t] == k5*ES[t]*Ox[t],
  EE[0] == 1.20*10^-6,
  ES[0]== 0,
  EOx[0]== 0,
  Ox[0]==4.0*10^-5,
  P[0]== 0},P,{t,0,totaltime},{k1,k2,k3,k4,k5}];

Clear[EE,ES,EOx,S,Ox,P,k1,k2,k3,k4,k5];
Needs["NonlinearRegression`"]
NonlinearRegress[data1,model[k1,k2,k3,k4,k5][t],{{k1,1*10^9},{k2,4000},{k3,3*10^7},{k4,
2},{k5,9*10^6}},t]
```

Modeling the Conversion of Cpd I-[Fe^{IV}=O, $\pi^{+\cdot}$] to Cpd I*-[Fe^{IV}-OH, $aa^{+\cdot}$] During Catalysis. To explore the possible role of Cpd I-[Fe^{IV}=O, $\pi^{+\cdot}$], Mathematica was used to model the mechanism of Figure 1A with the inclusion of the conversion of Cpd I-[Fe^{IV}=O, $\pi^{+\cdot}$] to Cpd I*-[Fe^{IV}-OH, $aa^{+\cdot}$]. The initial concentrations were the same as Figure 5. In the model EE = LPO, ES = LPO-SCN, Ox = H_2O_2, EOx = LPO-H_2O_2 = Cpd I*, S = SCN^-, and P = $OSCN^-$. The rate constants are taken from Table 1 and (*3*). The results of Figure S15 predict the majority of the steady-state species formed is LPO-[Fe^{III}], with a small amount of Cpd I-[Fe^{IV}=O, $\pi^{+\cdot}$]. Also, the production of $OSCN^-$ is expected to be pseudo-first-order (cf. Figure 4B).

```
Clear[EE, EOx, EOH, Ox, S, P, t, k1, k2, k3];

equations = {
```

```
  EE'[t] == -k1*EE[t]*Ox[t] + k2*EOx[t]*S[t],
  EOx'[t] == k1*EE[t]*Ox[t] - k2*EOx[t]*S[t],
  Ox'[t] == -k1*EE[t]*Ox[t],
  S'[t] == -k2*EOx[t]*S[t],
  P'[t] == k2*EOx[t]*S[t],
  EOH'[t] == k3*EOH[t],
  EE[0] == EE0,
  EOx[0] == 0,
  EOH[0] == 0,
  Ox[0] == Ox0,
  S[0] == S0,
  P[0] == 0
  };

k1 = 1.1*10^7;
k2 = 2*10^8;
k3 = 2;
EE0 = 1.2*10^-6;
S0 = 5.2*10^-5;
Ox0 = 4*10^-5;

timeframe = 0.5;

answer = NDSolve[
  equations, {EE[t], EOx[t], EOH[t], S[t], P[t], Ox[t]}, {t, 0,
   timeframe}];

EE[t_] = EE[t] /. answer;
EOx[t_] = EOx[t] /. answer;
EOH[t_] = EOH[t] /. answer;
S[t_] = S[t] /. answer;
P[t_] = P[t] /. answer;
Ox[t_] = Ox[t] /. answer;
```

$[SCN^-]$ = 0.1-16 mM
[LPO] = 0.1-8 μM
[TNB] = 200 μM
in 0.1 M pH 7 Pi

$[H_2O_2]$ = 80 μM
in water

$[SCN^-]$ = 0.1-8 mM
[LPO] = 0.05-4 μM
[TNB] = 100 μM
$[H_2O_2]$ = 40 μM
in 0.05 M pH 7 Pi

Figure S1. Reaction mixing scheme for the single mixing stopped-flow experiment to observe the rate of the LPO-catalyzed oxidation of SCN^- by H_2O_2 at pH 7.

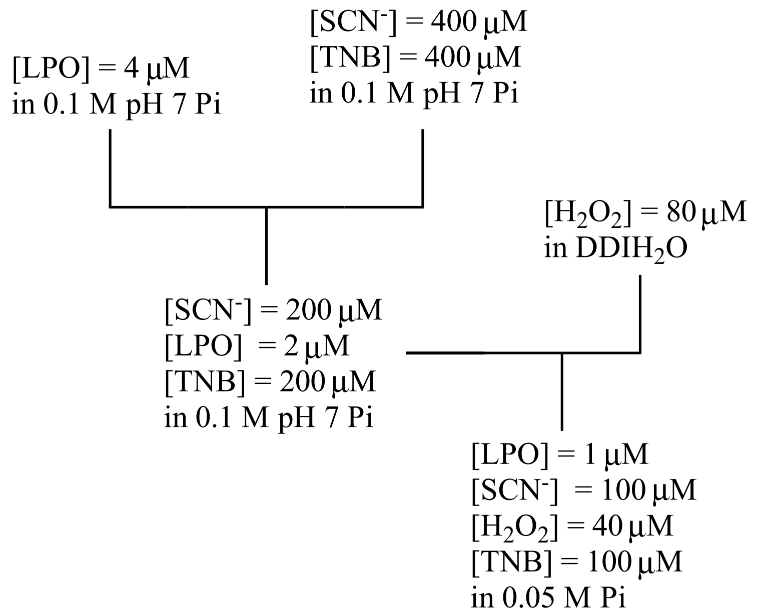

Figure S2. Reaction mixing scheme for the double mixing stopped-flow experiment to observe the effect of mixing LPO and SCN^- in the first mixing cycle prior to the addition of H_2O_2 on the LPO-catalyzed oxidation of SCN^- by H_2O_2 at neutral pH. This mixing scheme was used to produce the data in Figure 3A.

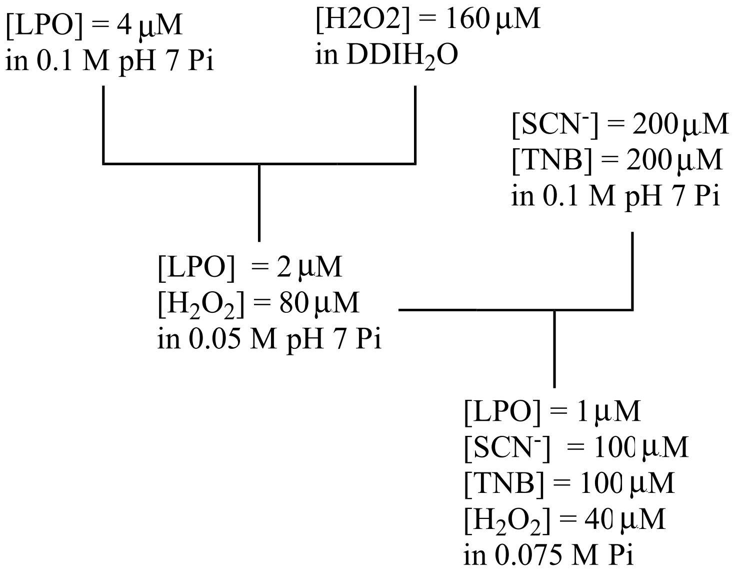

Figure S3. Reaction mixing scheme for the double mixing stopped-flow experiment to observe the effect of mixing LPO and H_2O_2 in the first mixing cycle prior to the addition of SCN^- on the LPO-catalyzed oxidation of SCN^- by H_2O_2 at neutral pH. This mixing scheme was used to produce the data in Figure 3B.

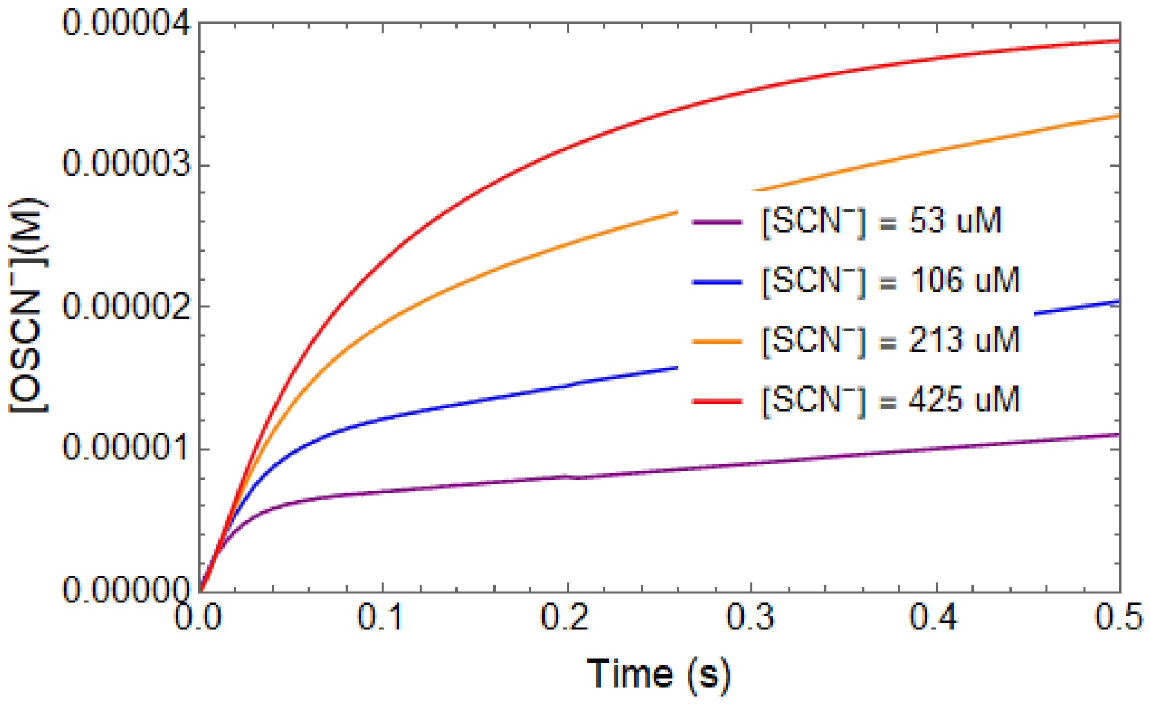

Figure S4. Isolated view of the pre-steady-state reaction observed in Figure 2 (left) for the LPO-catalyzed oxidation of [SCN$^-$] by H_2O_2 under low [SCN$^-$] conditions. Post-mixing concentrations were [LPO] = 1.2 μM, [H_2O_2] = 40 μM, [TNB] = 100 μM, and [SCN$^-$] = 52, 106, 213, and 425 μM. Notably, the initial reaction rates do not vary and the number of enzyme turnovers that occur during the pre-steady-state reaction increase with increasing [SCN$^-$].

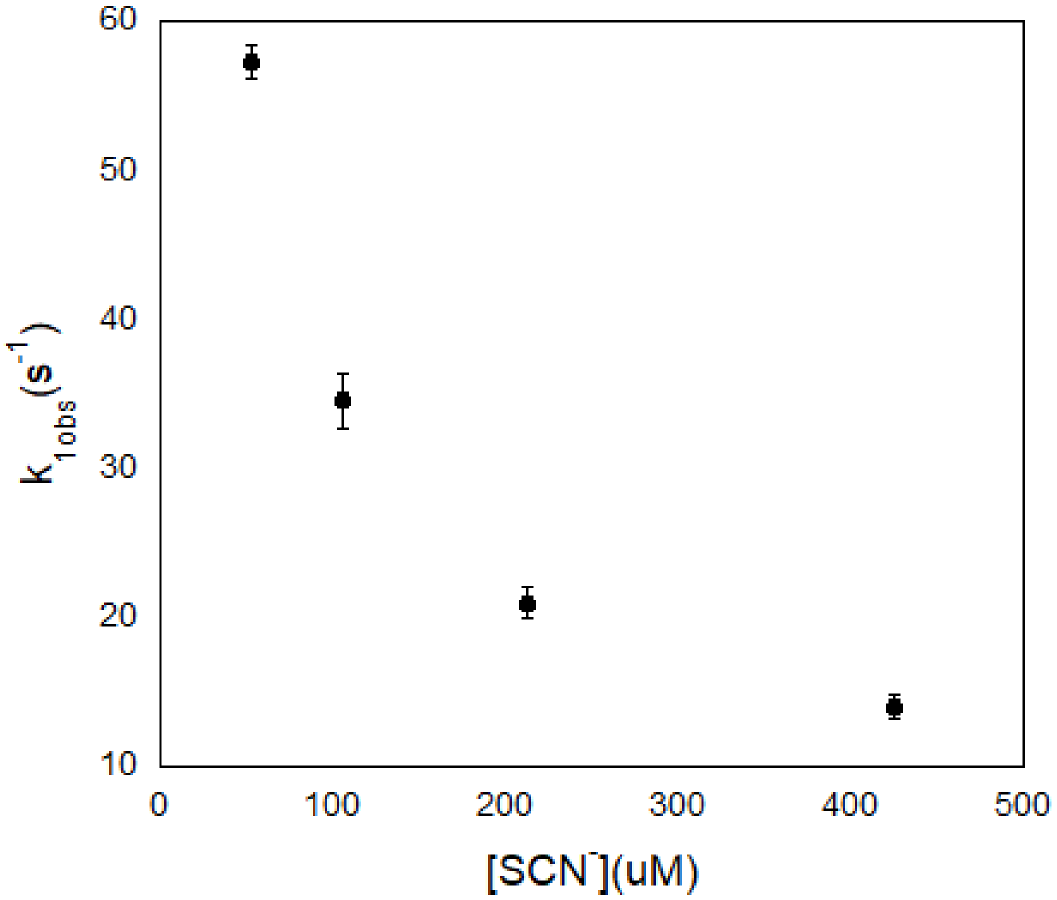

Figure S5. Observed first-order rate constants for the pre-steady-state reaction in the LPO-catalyzed oxidation of SCN^- by H_2O_2. Post-mixing concentrations were [LPO] = 1.2 µM, [H_2O_2] = 40 µM, [TNB] = 100 µM, and [SCN^-] = 52, 106, 213, and 425 µM. The estimated error shown by the error bars for the individual rate constants is for a least-squares fit of an average of five kinetic traces and has been multiplied by a factor of 10 so that the error bars would be visible on the plot.

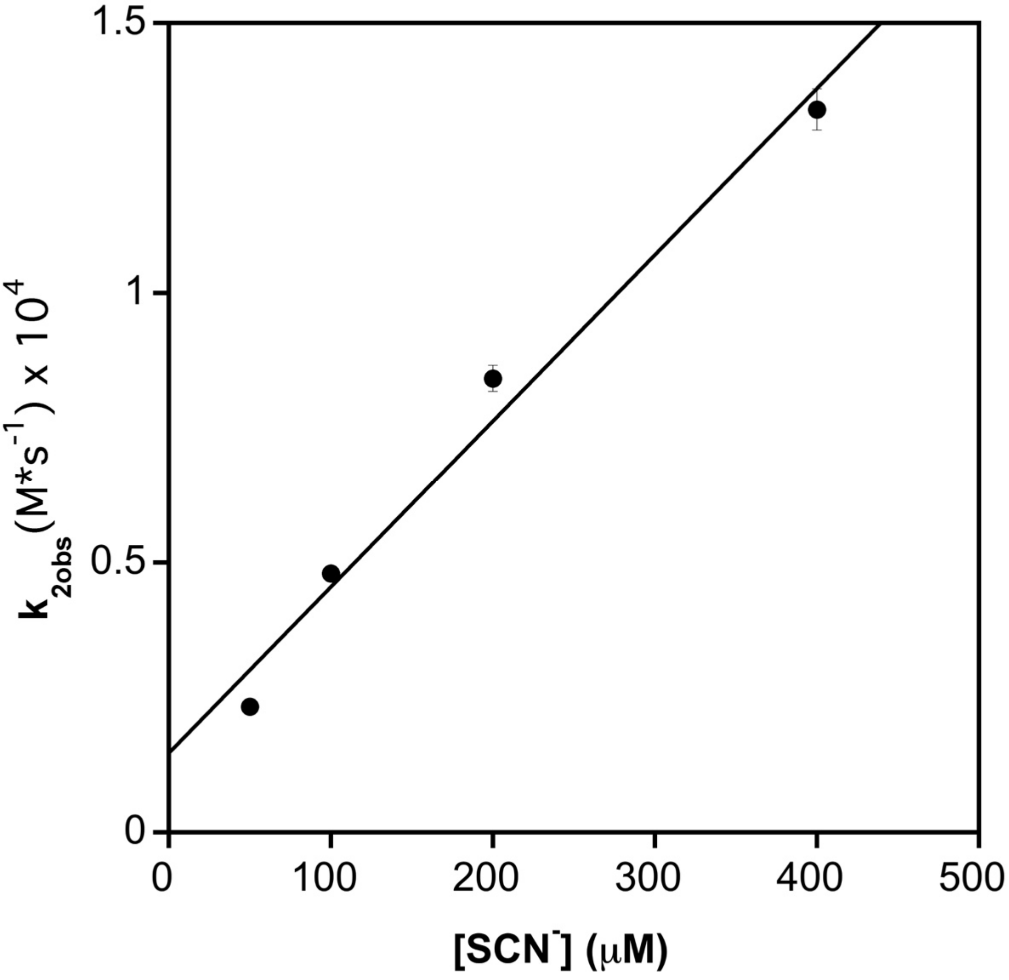

Figure S6. Effect of varying [SCN^-] on the rate of the steady-state reaction during the LPO-catalyzed oxidation of SCN^- by H_2O_2. Post-mixing concentrations were [LPO] = 0.1 μM, [H_2O_2] = 40 μM, [TNB] = 100 μM, and [SCN^-] = 50, 100, 200, and 400 μM. The estimated error shown by the error bars for the individual rate constants is for a least-squares fit of an average of five kinetic traces and has been multiplied by a factor of 10 so that the error bars would be visible on the plot.

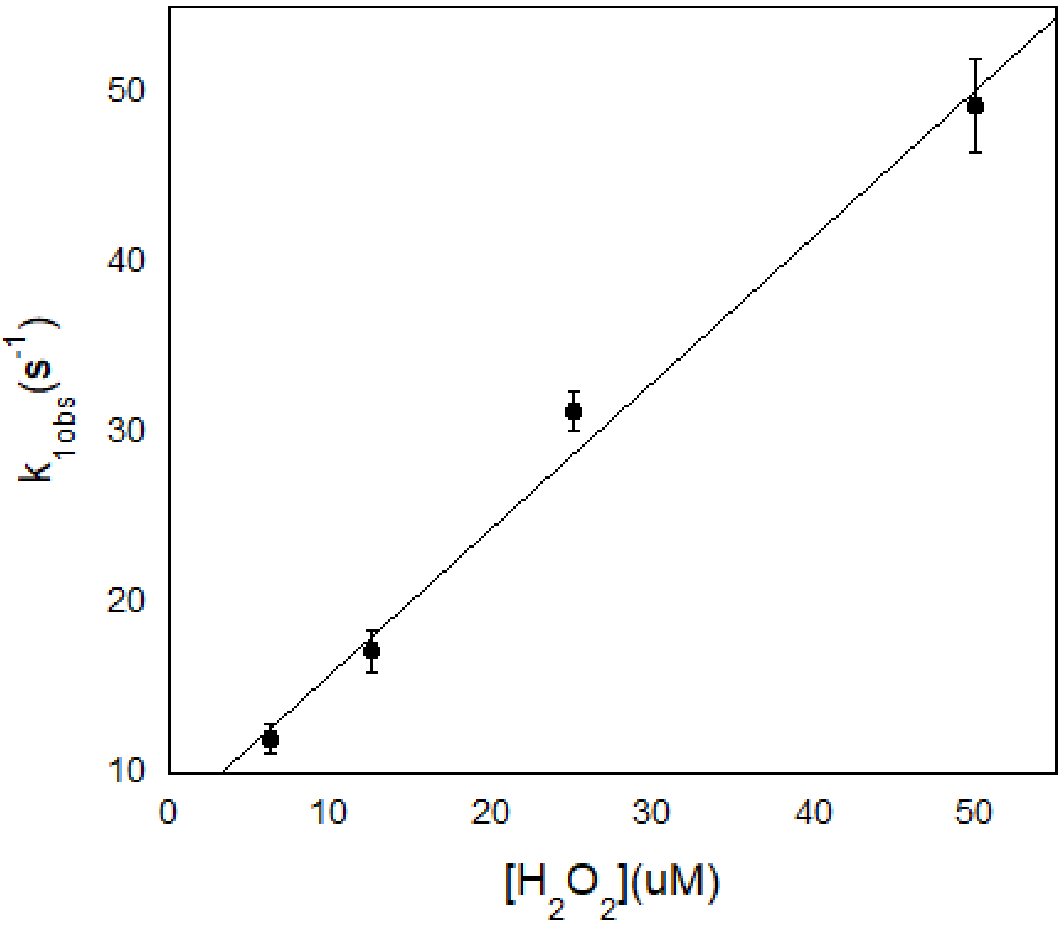

Figure S7. Effect of [H_2O_2] on k_{1obs} for the LPO-catalyzed oxidation of SCN^- by H_2O_2 under conditions of low concentration of [SCN^-]. Post-mixing concentrations were [LPO] = 0.5 µM, [SCN^-] = 100 µM, [TNB] = 100 µM, and [H_2O_2] = 6.25, 12.5, 25, and 50 µM. The estimated error shown by the error bars for the individual rate constants is for a least-squares fit of an average of five kinetic traces and has been multiplied by a factor of 10 so that the error bars would be visible on the plot.

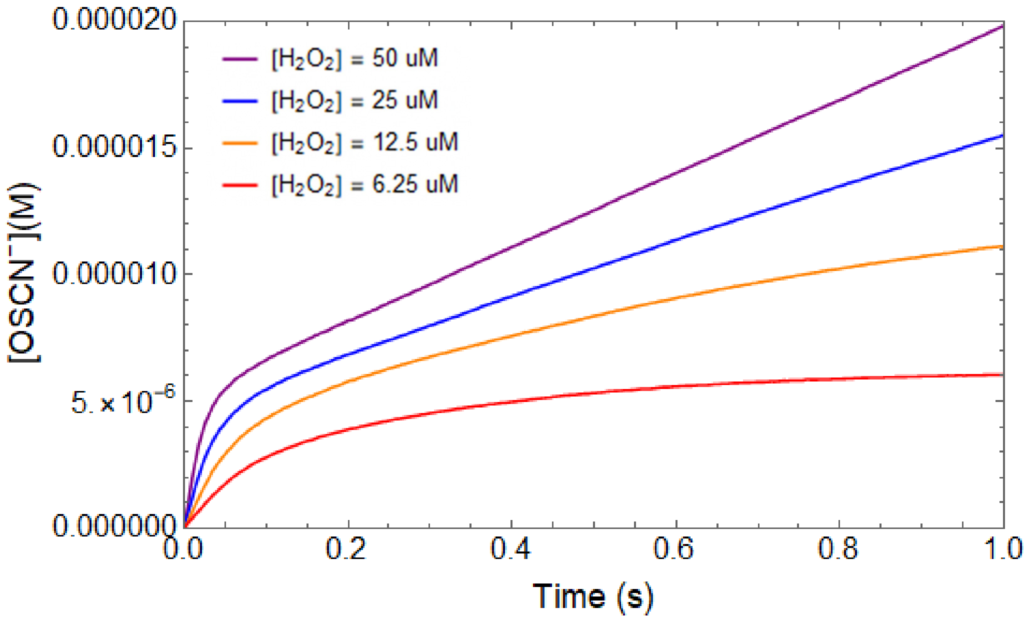

Figure S8. Kinetic traces of the pre-steady-state reaction observed during the LPO-catalyzed oxidation of SCN^- by H_2O_2 as $[H_2O_2]$ was varied. Post-mixing concentrations were $[SCN^-]$ = 100 μM, [LPO] = 0.5 μM, [TNB] = 100 μM, and $[H_2O_2]$ = 6.25, 12.5, 25, and 50 μM.

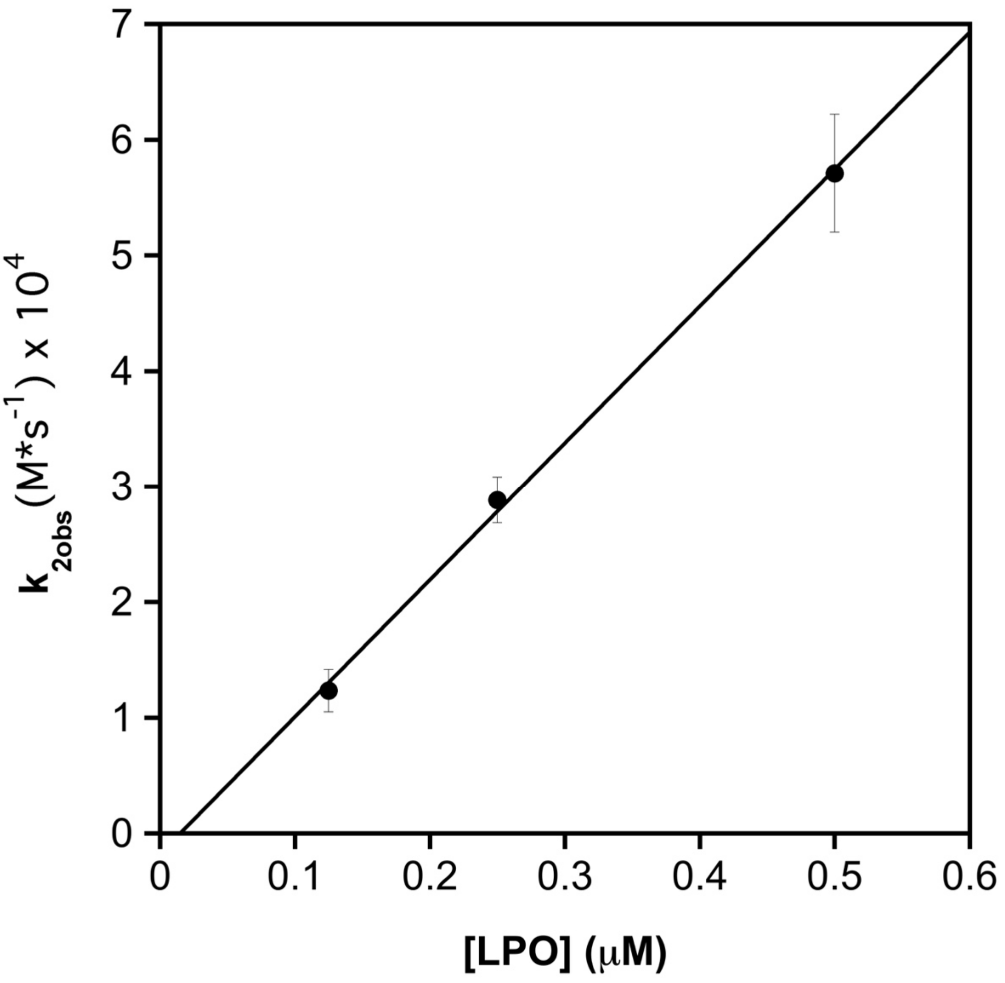

Figure S9. Steady-state reaction rate dependence on [LPO] for the LPO-catalyzed oxidation of SCN^- by H_2O_2. Post-mixing concentrations were [SCN^-] = 200 μM, [H_2O_2] = 40 μM, [TNB] = 100 μM, and [LPO] = 0.125, 0.25, and 0.5 μM. The estimated error shown by the error bars for the individual rate constants is for a least-squares fit of an average of five kinetic traces.

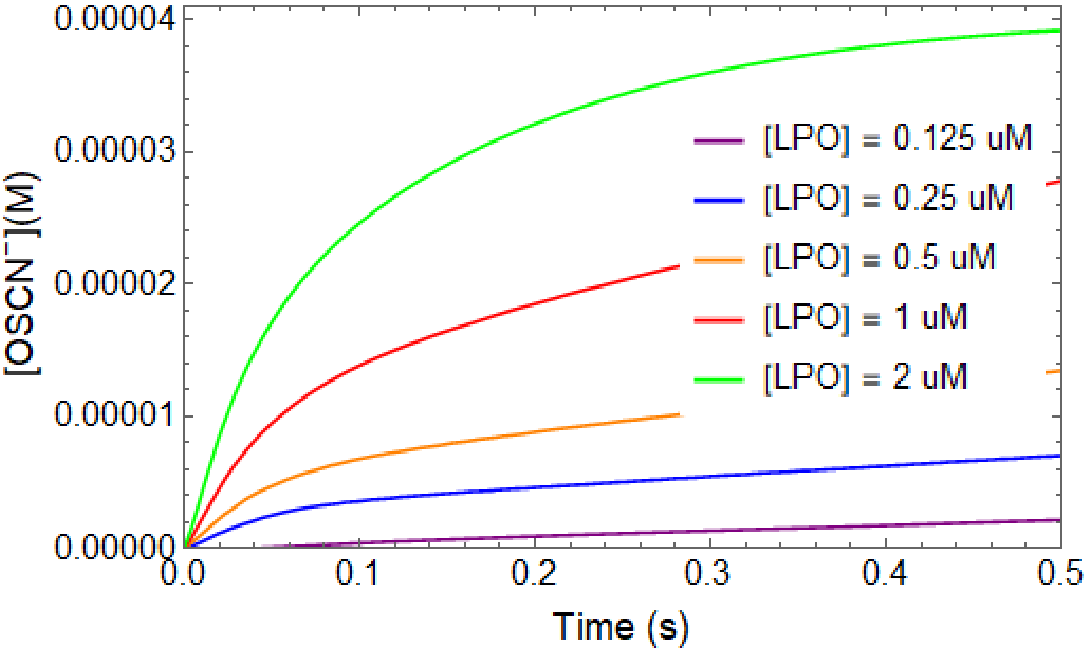

Figure S10. Kinetic traces of pre-steady-state reaction of the LPO-catalyzed oxidation of SCN^- by H_2O_2 as [LPO] is varied. Post-mixing concentrations were [SCN^-] = 200 µM, [H_2O_2] = 40 µM, [TNB] = 100 µM, and [LPO] = 0.125-2 µM

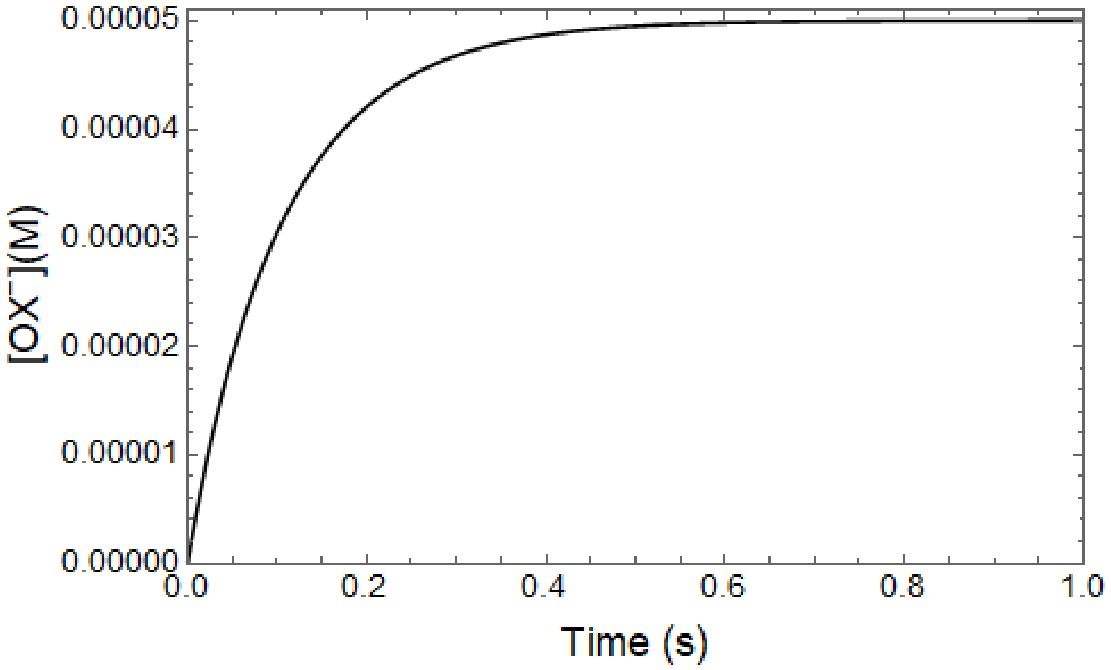

Figure S11. Simulated trace of [$OSCN^-$] for the proposed mechanism for the LPO-catalyzed oxidation of SCN^- by H_2O_2 under conditions of high [SCN^-].

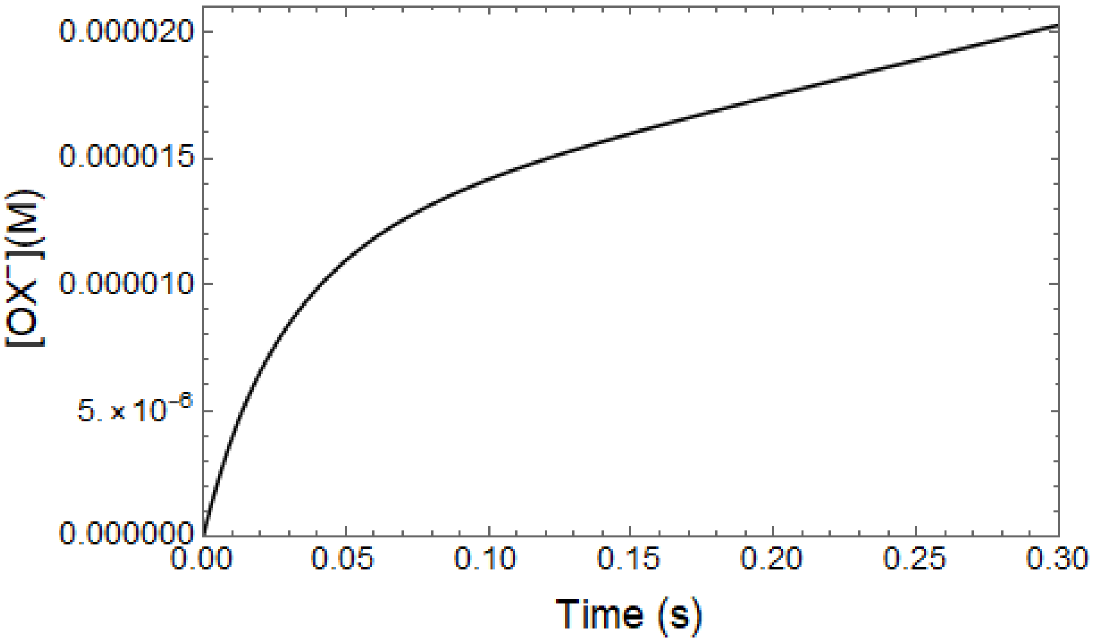

Figure S12. Simulated trace of [OSCN$^-$] during the pre-steady-state reaction for the proposed mechanism for the LPO-catalyzed oxidation of SCN$^-$ by H_2O_2 under conditions of low [SCN$^-$].

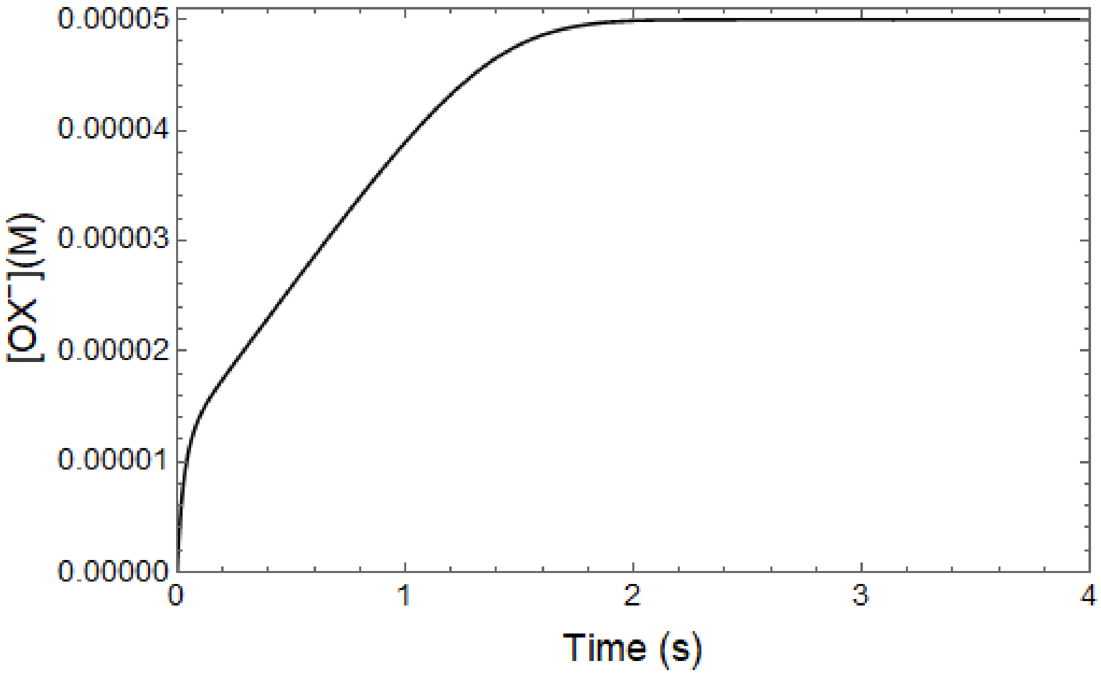

Figure S13. Simulated trace of [OSCN$^-$] during the reaction for the proposed mechanism for the LPO-catalyzed oxidation of SCN$^-$ by H_2O_2 under conditions of low [SCN$^-$].

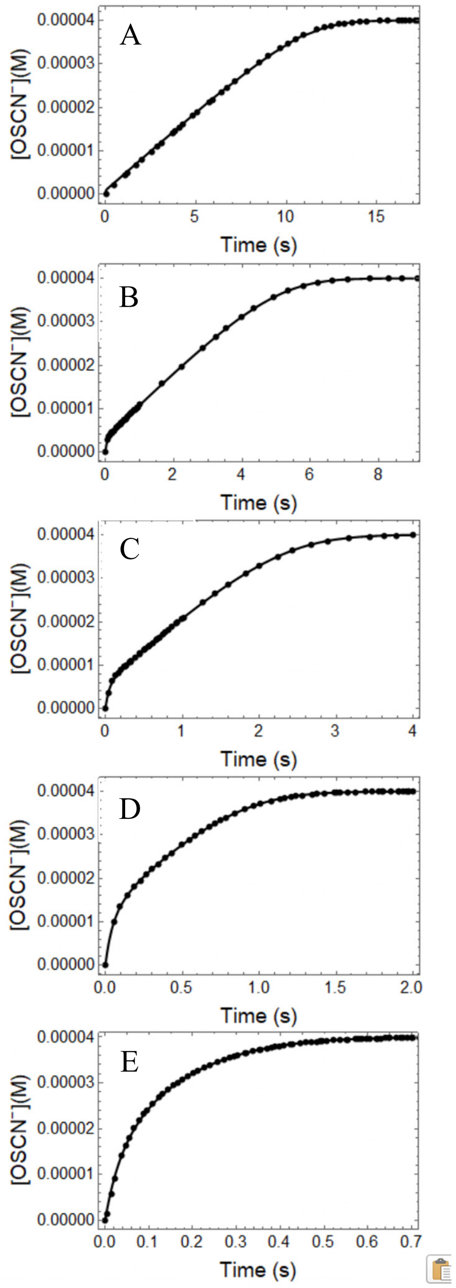

Figure S14. Fitted kinetic traces for the LPO-catalyzed oxidation of SCN^- by H_2O_2 as [LPO] was varied. Post-mixing concentrations were [SCN^-] = 200 μM, [H_2O_2] = 40 μM, [TNB] = 100 μM, and [LPO] = 0.125, 0.25, 0.5, 1, and 2 μM for A, B, C, D, and E, respectively. Rate constants produced by these fits are given in Table S8

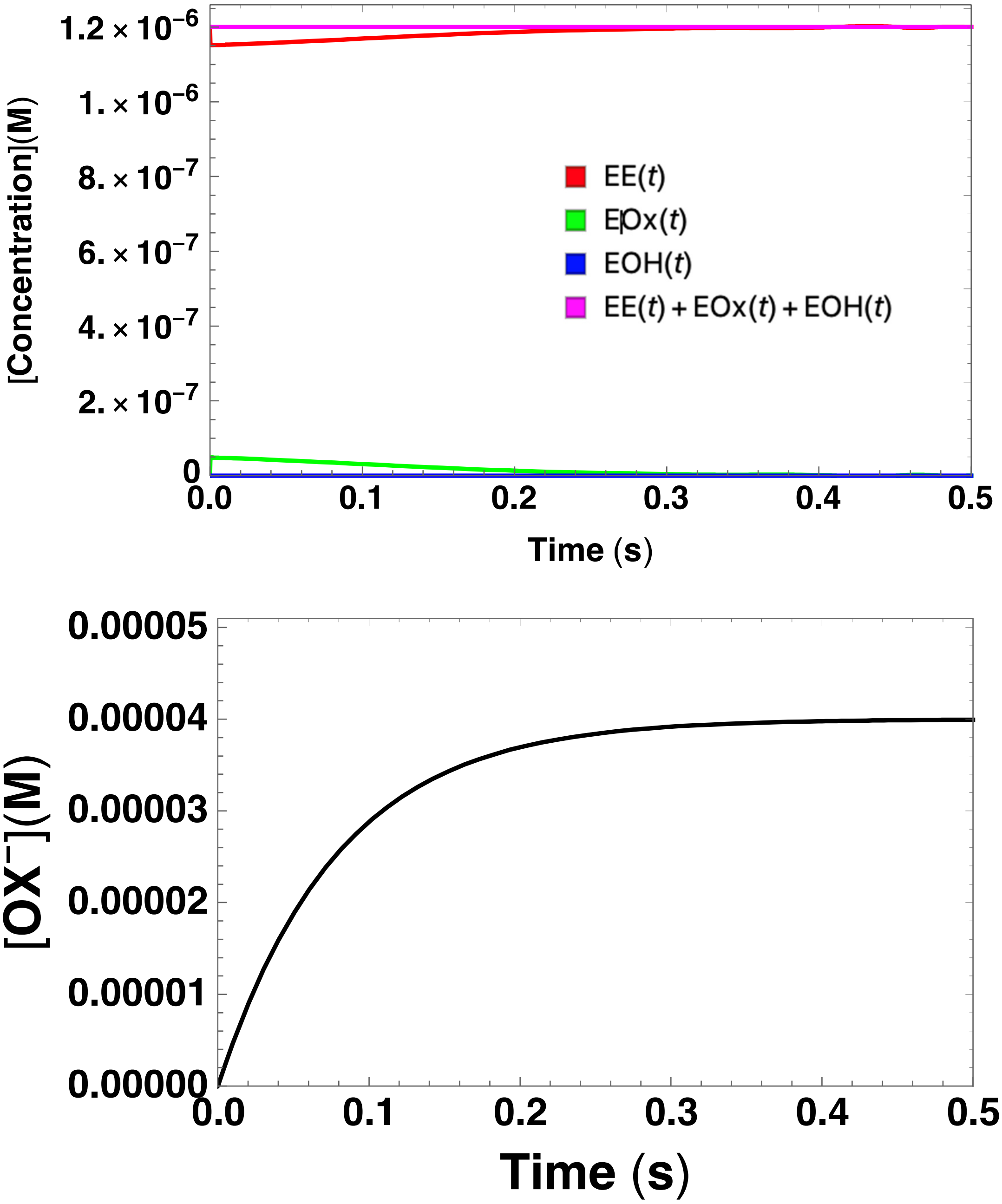

Figure S15. Simulated concentrations during the LPO-catalyzed oxidation of SCN^- by H_2O_2 for the mechanism of Figure 1A with the additional known step for the conversion of Cpd I to Cpd I*: Red = LPO-[Fe^{III}], Green = Cpd I, Blue = Cpd I* (not observed), Black = $OSCN^-$, $k_1 = 1.1 \times 10^7$ $M^{-1}s^{-1}$, $k_2 = 2 \times 10^8$ $M^{-1}s^{-1}$, $k_3 = 2$ s^{-1}, $[LPO]_0 = 1.2 \times 10^{-6}$ M, $[H_2O_2]_0 = 4 \times 10^{-5}$ M. $[SCN^-]_0 = 5.2 \times 10^{-5}$ M. This simulation assumes the [SCN^-] remains constant, as expected for the stoichiometry of the TNB assay.

Table S1. Observed rate of the LPO-catalyzed oxidation of SCN^- by H_2O_2 as a function of $[H_2O_2]$.[a]

$[H_2O_2]$ (μM)	k_{obs} (s^{-1})[b]	Model Predicted $k_{obs\text{-}sim}$ (s^{-1})
7.5	6.56(4)	11.00
15	5.65(3)	11.00
30	7.02(8)	11.00
Average	6.4(7)[c]	11.00

[a]A single mixing experiment with constant post-mixing concentrations of [LPO] = 1 μM, [SCN^-] = 4 mM, [TNB] = 70 μM, and [H_2O_2] = 7.5-30 μM.

[b]The estimated error, given by the parenthetical digits, for the individual rate constants is for a least-squares fit of an average of five kinetic traces.

[c]The average error was determined by the standard deviation of the individual fits.

Table S2. Observed rate of the LPO-catalyzed oxidation of SCN^- by H_2O_2 as a function of $[SCN^-]$.[a]

$[SCN^-]$ (mM)	k_{obs} (s^{-1})[b]	Model Predicted $k_{obs\text{-}sim}$ (s^{-1})
1.2	6.29(3)	11.00
2.1	7.96(2)	11.00
4.3	8.839(8)	11.00
8.6	8.93(1)	11.00
Average[c]	–	11.00

[a]Single mixing experiment with constant post-mixing concentrations of [LPO] = 1 μM, $[H_2O_2]$ = 40 μM, and [TNB] = 100 μM.

[b]The estimated error, given by the parenthetical digits, for the individual rate constants is for a least-squares fit of an average of five kinetic traces.

[c]The average of the experimentally observed rate constants was not calculated due to systematic error.

Table S3. Observed initial rates of the LPO-catalyzed oxidation of SCN^- by H_2O_2 as a function of $[SCN^-]$.[a]

$[SCN^-]$ (μM)	k_{obs} ($M \cdot s^{-1}$)[b]
53	$1.00(1)x10^{-3}$
106	$1.020(8)x10^{-3}$
213	$1.095(4)x10^{-3}$
425	$1.124(7)x10^{-3}$
Average	$1.06(5)x10^{-3}$

[a]Single mixing experiment with constant post-mixing concentrations of [LPO] = 1.2 μM, $[H_2O_2]$ = 40 μM, and [TNB] = 100 μM.

[b]The estimated error, given by the parenthetical digits, for the individual rate constants is for a least-squares fit of an average of five kinetic traces.

Table S4. Effect of varying [H_2O_2] on the rate of the steady-state reaction of the LPO-catalyzed oxidation of SCN^- by H_2O_2.[a]

[H_2O_2] (μM)	k_{2obs} ($M \cdot s^{-1}$)[b]
5	3.00(1)x10^{-6}
10	4.01(1)x10^{-6}
20	4.54(1)x10^{-6}
40	4.65(1)x10^{-6}
Average	4.0(6)x10^{-6}

[a]The post-mixing concentrations were [LPO] = 0.1 μM, [SCN^-] = 100 μM, [TNB] = 100 μM, and [H_2O_2] = 5, 10, 20, and 40 μM.

[b]The estimated error, given by the parenthetical digits, for the individual rate constants is for a least-squares fit of an average of five kinetic traces.

[c]The average error of the model predicted rate constants was determined by the standard deviation of the individual fits.

Table S5. Effect of varying [LPO] on the rate of the pre-steady-state reaction for the LPO-catalyzed oxidation of SCN^- by H_2O_2.[a]

[LPO] (μM)	k_{1obs}(s^{-1})[b]
0.25	23.5(1)
0.5	23.07(9)
1	20.7(1)
Average	22(2)[c]

[a]Post-mixing concentrations were [SCN^-] = 200 μM, [H_2O_2] = 40 μM, [TNB] = 100 μM, and [LPO] = 0.25, 0.5, and 1 μM.

[b]The estimated error, given by the parenthetical digits, for the individual rate constants is for a least-squares fit of an average of five kinetic traces.

[c]The average error was calculated by the standard deviation of the individual fits.

Table S6. Fitted rate constants for the proposed mechanism of the LPO-catalyzed oxidation of SCN^- by H_2O_2 as $[SCN^-]$ was varied.[a]

$[SCN^-]$(μM)	k_1 ($M^{-1}\cdot s^{-1}$)[b]	k_{-1} (s^{-1})[b]	k_2 ($M^{-1}\cdot s^{-1}$)[b]	k_{-2} (s^{-1})[b]	k_3 ($M^{-1}\cdot s^{-1}$)[b]
53	1.31(7)x10^9	4.6(3)x10^3	2.23(3)x10^7	1.556(4)	8.62(3)x10^6
106	1.33(3)x10^9	3.9(3)x10^3	3.04(1) x10^7	1.760(3)	9.24(3)x10^6
213	1.30 x10^9(fixed)	3.93x10^3(fixed)	2.81(2) x10^7	1.68(1)	9.27(4)x10^6
425	1.30x10^9(fixed)	3.93x10^3(fixed)	2.65(3) x10^7	1.72(fixed)	9.44(4) x10^6
Average[c]	1.32(1)x10^9	4.3(5)x10^3	2.7(3)x10^7	1.7(1)	9.1(4)x10^6

[a]The post-mixing concentrations were [LPO] = 1.2 μM, $[H_2O_2]$ = 40 μM, [TNB] = 100 μM, and $[SCN^-]$ = 53, 106, 213, and 425 μM.

[b]The estimated error, given by the parenthetical digits, for the individual rate constants is for a least-squares fit.

[c]The average error was calculated by the standard deviation of the individual fits.

Table S7. Fitted rate constants for the proposed mechanism of the LPO-catalyzed oxidation of SCN^- by H_2O_2 as $[H_2O_2]$ was varied.[a]

$[H_2O_2]$(μM)	k_1 ($M^{-1}\cdot s^{-1}$)[b]	k_{-1} (s^{-1})[b]	k_2 ($M^{-1}\cdot s^{-1}$)[b]	k_{-2} (s^{-1})[b]	k_3 ($M^{-1}\cdot s^{-1}$)[b]
50	1.58(2)x10^9	3.5(3)x10^3	5.91(3x10^{-3})x10^7	2.58(2)	1.84(4)x10^7
25	1.8(4)x10^9	3.2(7)x10^3	4.8(4)x10^7	2.14(2)	1.110(7)x10^7
12.5	1.65x10^9(fixed)	3.30x10^3(fixed)	6.4(1)x10^7	2.30(fixed)	1.44(2)x10^7
6.25	1.65x10^9(fixed)	3.30x10^3(fixed)	4.9(2)x10^7	2.30(fixed)	1.330(5)x10^7
Average[c]	1.7(1)x10^9	3.3(2)x10^3	5.5(8)x10^7	2.4(3)	1.4(3)x10^7

[a]The post-mixing concentrations were $[SCN^-]$ = 100 μM, [LPO] = 0.5 μM, [TNB] = 100 μM, and $[H_2O_2]$ = 6.25, 12.5, 25, and 50 μM.

[b]The estimated error, given by the parenthetical digits, for the individual rate constants is for a least-squares fit.

[c]The average error was calculated by the standard deviation of the individual fits.

Table S8. Fitted rate constants for the proposed mechanism of the LPO-catalyzed oxidation of SCN^- by H_2O_2 as [LPO] was varied.[a]

[LPO](μM)	k_1 ($M^{-1}\cdot s^{-1}$)[b]	k_{-1} (s^{-1})[b]	k_2 ($M^{-1}\cdot s^{-1}$)[b]	k_{-2} (s^{-1})[b]	k_3 ($M^{-1}\cdot s^{-1}$)[b]
0.125	$1.20x10^9$(fixed)	$4.20x10^3$	$2.42x10^7$(fixed)	1.98(1)	$7.39(2)x10^6$
0.25	$1.2(2)x10^9$	$4.2(4)x10^3$	$2.5(2)x10^7$	2.17(1)	$7.30(3)x10^6$
0.5	$1.17(2)x10^9$	$4.2(7)x10^3$	$2.5(4)x10^7$	2.35(1)	$7.32(5)x10^6$
1	$1.20x10^9$(fixed)	$4.20x10^3$(fixed)	$2.27(1)x10^7$	2.39(1)	$7.56(2)x10^6$
2	$1.20x10^9$(fixed)	$4.20x10^3$(fixed)	$1.79(1)x10^7$	2.30(fixed)	$7.19(2)x10^6$
Average[c]	$1.20(4)x10^9$	$4.18(3)x10^3$	$2.3(3)x10^7$	2.2(2)	$7.4(1)x10^6$

[a]The post-mixing concentrations were [SCN^-] = 200 μM, [H_2O_2] = 40 μM, [TNB] = 100 μM, and [LPO] = 0.125, 0.25, 0.5, 1, and 2 μM.

[b]The estimated error, given by the parenthetical digits, for the individual rate constants is for a least-squares fit.

[c]The average error was calculated by the standard deviation of the individual fits.
